# Expansion of CD4+ cytotoxic T lymphocytes with specific gene expression patterns may contribute to suppression of tumor immunity in oral squamous cell carcinoma: single-cell analysis and *in vitro* experiments

**DOI:** 10.3389/fimmu.2023.1305783

**Published:** 2023-11-23

**Authors:** Hu Chen, Junsei Sameshima, Shiho Yokomizo, Tomoki Sueyoshi, Haruki Nagano, Yuka Miyahara, Taiki Sakamoto, Shinsuke Fujii, Tamotsu Kiyoshima, Thomas Guy, Seiji Nakamura, Masafumi Moriyama, Naoki Kaneko, Shintaro Kawano

**Affiliations:** ^1^ Section of Oral and Maxillofacial Oncology, Division of Maxillofacial Diagnostic and Surgical Sciences, Faculty of Dental Science, Kyushu University, Fukuoka, Japan; ^2^ Laboratory of Oral Pathology, Division of Maxillofacial Diagnostic and Surgical Sciences, Faculty of Dental Science, Kyushu University, Fukuoka, Japan; ^3^ Ragon Institute of MGH, MIT and Harvard, Massachusetts General Hospital, Harvard Medical School, Boston, MA, United States; ^4^ Faculty of Dental Science, Kyushu University, Fukuoka, Japan

**Keywords:** oral squamous cell carcinoma, CD4+ cytotoxic T lymphocytes, CTLA-4, T cell dysfunction, CXCL13, double negative B cells

## Abstract

**Background:**

Cancer immunotherapy targeting CD8^+^ T cells has made remarkable progress, even for oral squamous cell carcinoma (OSCC), a heterogeneous epithelial tumor without a substantial increase in the overall survival rate over the past decade. However, the therapeutic effects remain limited due to therapy resistance. Thus, a more comprehensive understanding of the roles of CD4^+^ T cells and B cells is crucial for more robust development of cancer immunotherapy.

**Methods:**

In this study, we examined immune responses and effector functions of CD4^+^ T cells, CD8^+^ T cells and B cells infiltrating in OSCC lesions using single-cell RNA sequencing analysis, T cell receptor (TCR) and B cell receptor (BCR) repertoire sequencing analysis, and multi-color immunofluorescence staining. Finally, two Kaplan-Meier curves and several Cox proportional hazards models were constructed for the survival analysis.

**Results:**

We observed expansion of CD4^+^ cytotoxic T lymphocytes (CTLs) expressing granzymes, which are reported to induce cell apoptosis, with a unique gene expression patterns. CD4^+^ CTLs also expressed CXCL13, which is a B cell chemoattractant. Cell–cell communication analysis and multi-color immunofluorescence staining demonstrated potential interactions between CD4^+^ CTLs and B cells, particularly IgD^-^ CD27^-^ double negative (DN) B cells. Expansion of CD4^+^ CTLs, DN B cells, and their contacts has been reported in T and B cell-activated diseases, including IgG4-related disease and COVID-19. Notably, we observed upregulation of several inhibitory receptor genes including CTLA-4 in CD4^+^ CTLs, which possibly dampened T and B cell activity. We next demonstrated comprehensive delineation of the potential for CD8^+^ T cell differentiation towards dysfunctional states. Furthermore, prognostic analysis revealed unfavorable outcomes of patients with a high proportion of CD4^+^ CTLs in OSCC lesions.

**Conclusion:**

Our study provides a dynamic landscape of lymphocytes and demonstrates a systemic investigation of CD4^+^ CTL effects infiltrating into OSCC lesions, which may share some pathogenesis reported in severe T and B cell-activated diseases such as autoimmune and infectious diseases.

## 1 Introduction

Cancer immunotherapy has revolutionized the treatment environment. The two principal treatment modalities are immune checkpoint inhibitor therapy and adoptive cell therapy represented by chimeric antigen receptor-T cell therapy. Both therapies share a mutual objective, namely eradicating cancer cells and improving prognosis by activating cytotoxic T lymphocytes (CTLs), particularly the CD8^+^ CTL subpopulation. These therapies have demonstrated substantial efficacy in patients who are refractory to existing therapies.

Oral squamous cell carcinoma (OSCC) is a heterogeneous epithelial tumor without a substantial increase in the overall survival rate over the past decade, largely because of its high propensity for local invasion and recurrence (1). Although evidence supports adjuvant chemoradiation for high-risk individuals, recurrence remains prevalent and disease-related death is considerable, even at early time points. Similar to other solid tumors, programmed cell death protein 1 (PD-1) inhibitors nivolumab and pembrolizumab have been approved for patients with recurrent/metastatic head and neck squamous cell carcinomas including OSCC. Furthermore, the application of neoadjuvant immunotherapy, nivolumab, and cytotoxic T-lymphocyte-associated protein 4 (CTLA-4) inhibitor ipilimumab prior to surgical intervention has recently been recognized to be a feasible treatment (2). Several reports on the influence of human leukocyte antigen (HLA) genetic factors and some antigenic peptides recognized by CTLs in OSCC suggest the effectiveness of these immunotherapies. However, regardless of various ongoing efforts to improve treatment outcomes, the therapeutic effect is limited because a fraction of patients remains non-responsive or quickly become resistant, and a certain rate of hyper-progression occurs (3). Consequently, while current studies about tumor immunity have primarily focused on CD8^+^ T cells and tumor cells, especially in OSCC, a more comprehensive understanding that is not confined to these cell types is crucial for more robust development of cancer immunotherapy.

In addition to CD8^+^ T cells, the pivotal role of CD4^+^ T cells in instigating and maintaining effective tumor immunity has been gaining recognition, even in the context of cancer immunotherapies tailored to induce a CD8^+^ CTL response (2). Recently, Kruse et al. demonstrated that CD4^+^ effector T cells are inclined to infiltrate the invasive front of major histocompatibility complex (MHC) class I-deficient tumors that may escape eradication mediated by CD8^+^ CTLs (4). This substantial discovery elucidates how CD4^+^ T cells indirectly eradicate tumor cells through collaboration with tumor-associated myeloid cells and stimulation of innate immune cells. In fact, several studies have provided evidence associating expression of MHC class II in tumor cells with favorable outcomes of many cancer types (5–7).

Apart from T cells, mounting evidence suggests that B cells infiltrating tumor lesions and antibodies produced by plasma cells play a crucial role in tumor immunity. This is notably supported by Roei et al. who comprehensively demonstrated that tumor-reactive antibodies are produced by both germline-encoded autoreactive antibody-secreting cells (ASCs) and ASCs which underwent somatic hypermutations (SHMs) and affinity maturation (8). Despite the controversy surrounding their role in tumor immunity, a comprehensive understanding of B cells and the antibodies they produce remains indispensable for OSCC treatment.

In this study, we found that specific subsets of CD4^+^ T cells, notably CD4^+^ CTLs, expand in OSCC by high-throughput sequencing and quantitative imaging. We have previously identified these subsets as pivotal contributors to the establishment of specific disease milieus such as systemic sclerosis, IgG4-related disease, and COVID-19 (9–11). While expansion of tumor neoantigen-specific CD8^+^ T cells, which contribute to cancer eradication, has been previously reported in other cancer types, we found that the sum of CD4^+^ T cells, B cells, and ASCs in OSCC was similar to CD8^+^ T cells, and CD4^+^ CTLs and activated B cells were the main CD45^+^ immune cells. Notably, CD4^+^ CTLs also expressed multiple kinds of inhibitory receptor (IR) genes, such as *PDCD1, CTLA4, LAG3*, and *HAVCR2*, and exhibited co-expression modules analogous to CD4^+^ regulatory T cells (Tregs), suggesting that they eliminate tumor cells via cytotoxic activity and act as immunosuppressor cells.

Furthermore, we found that CD4^+^ CTLs expressed C-X-C motif chemokine ligand 13 (CXCL13), a B cell chemoattractant, and physically interacted with activated B cells in tumor lesions. These activated B cells included a population of DN B cells that we have previously reported to be a disease-related B cell population that arises from the extrafollicular B cell response and are readily induced in the altered inflammatory milieu.

Our results indicate that CD4^+^ CTLs might be as pivotal to tumor immunity as CD8^+^ CTLs, and that CD4^+^ CTLs also interact with B cells through physical conjugation. This study provides a comprehensive landscape of the dynamics of T and B cells induced in OSCC lesions, potentially revealing some similarities with the pathogenesis of severe T and B cell-activated diseases such as autoimmune and infectious diseases.

## 2 Materials and methods

### 2.1 Study participants

The study included 23 patients with primary OSCC of the tongue treated at the Department of Oral and Maxillofacial Surgery, Kyushu University Hospital. Patients had been diagnosed from 2015 to 2017 at the Department of Kyushu University Hospital. The OSCC histological grade was determined by the World Health Organization’s classification system, and the tumor grade was determined by the TNM classification system. All included patients had a pathological diagnosis by biopsy and underwent scheduled excision surgery. A summary of OSCC patient information is presented in **Supplementary Table 1**. This study was approved by the Ethics Committee of Kyushu University Hospital (IRB number: 2021-265). Informed consent was obtained from each patient. All experiments were performed in accordance with the relevant guidelines and regulations.

### 2.2 Tissue homogenization and CD45^+^ cell isolation

All fresh tissue samples of OSCC were collected at the time of surgical resection. Excised tongue specimens from three OSCC patients (**Supplementary Table 1**, patients marked as ‘scRNA-seq’ in the Application column) were preserved in Tissue Storage Solution (130-100-008, Miltenyi Biotec) for subsequent processing. The tissue samples were transferred to gentleMACS C Tubes (130-093-237, Miltenyi Biotec) and cut into small pieces in RPMI-1640 with L-glutamine and phenol red (189-02025, FUJIFILM). Multi Tissue Dissociation Kit I (130-110-201, Miltenyi Biotec) and a gentleMACS Octo Dissociator with Heaters (130-096-427, Miltenyi Biotec) were employed to gently isolate cells from the tongue samples while preserving cell surface epitopes. Following homogenization for 1 hour, the tissue sample was passed through a 100 µm MACS SmartStrainer (130-098-463, Miltenyi Biotec) and transferred into a 5 mL Round Bottom Polystyrene Test Tube with Cell Strainer Snap Cap (352235, Falcon). After washing twice with EasySep Buffer (20144, STEMCELL), erythrocytes were eliminated using a Red Blood Cell Lysis Solution (130-094-183, Miltenyi Biotec).

For CD45^+^ cell isolation, the homogenized cell suspension was incubated with a PE anti-human CD45 antibody (304039, BioLegend) and FcR Blocking Reagent, human (130-059-901, Miltenyi Biotec) at room temperature (RT) for 15 min. Then, EasySep Human PE Positive Selection Kit II (17664, STEMCELL) was employed for positive selection of desired cells. Cells were labeled with anti-CD45^+^ antibody-coated magnetic particles and separated using an EasySep Magnet (18000, STEMCELL). After separation, the cells were preserved in CELLBANKER 1 plus (11912, ZENOGEN PHARMA) and stored at -80°C for subsequent analysis.

### 2.3 Single-cell RNA and TCR/BCR library preparation and sequencing

The number and viability of isolated CD45^+^ cells were assessed using a LUNA-FX7 Automated Cell Counter (L70001, Logos Biosystems). The cell concentration was adjusted to 10,000 single cells per sample. Subsequently, the cells were processed using a Chromium Single Cell 5′ Reagent Kit (v2) in the 10x Genomics Chromium instrument in accordance with the manufacturer’s protocol. To construct libraries, 10x Genomics Chromium Single Cell V(D)J v2 (GEX + human TCR/BCR) reagents were used. The prepared libraries were subjected to sequencing on an Illumina Hiseq platform with the following configuration: Read 1 (26 bp) + i7 index (8 bp) + i5 index (8 bp) + Read 2 (91 bp). Additionally, Hiseq 350M read/lane and two lanes per sample were used to ensure a sequence depth of 100,000 reads per cell.

### 2.4 Quality control and gene expression analysis

The raw sequencing data from Illumina Hiseq were processed to generate FASTQ files, which were then used to obtain single cell feature counts and V(D)J immune profiling analysis results using 10x Genomics Cell Ranger 6.0 software. Subsequent gene expression analysis and cell clustering were performed in the R 4.2.0 environment using the Seurat 4.3.0 package (12).

Before commencing the analysis, the gene expression data were first filtered based on the following criterion: cells exhibiting more than 15% mitochondrial RNA, fewer than 100 gene counts, and fewer than 200 gene types were considered lysed and removed from the dataset. The scRNA-seq gene expression data were normalized using the sctransform (SCT) method, and the respective expression data from the three patients were integrated to correct the batch effects. Dimensionality reduction of the gene expression matrix was performed using principle complement analysis (PCA), and visualization was achieved through uniform manifold approximation and projection (UMAP) dimensionality reduction (13).

A k-nearest neighbor graph was constructed based on the PCA results, and cell clusters were identified using Louvain algorithm (14). The leukocyte phenotypes were then assigned to these clusters using the following marker genes: *CD79A, MS4A1, CD19* for B cells; *CD79A, SDC1, XBP1, JCHAIN* for ASCs; *CD3E, CD4, IL7R* for CD4^+^ T cells; *CD3E, CD8A* for CD8^+^ T cells; *TPSAB1, CPA3* for mast cells; *CD14, CD68, AIF1* for macrophages; *IFIT1, MX2* for neutrophils; *NCAM1* for NK cells; *CD4, CLEC4C, IL3RA* for plasmacytoid dendritic cells (pDCs); *TRGV9, CD3E* for γδ T cells.

T cells and B cells were extracted and re-clustered using the pipeline mentioned above. Quality control measures were implemented to minimize the potential doublet in both T cells and B cells. For CD4^+^ T cells, any cells expressing *CD19, CD79A, MS4A1, CD8A, CD14* or *AIF1* were removed. Similarly, in CD8^+^ T cells, cells expressing *CD19, CD79A, MS4A1 CD4, CD14* and *AIF1* were excluded. Finally, for B cells, any cells expressing *CD3E, CD4, CD8A, CD14* or *AIF1* were removed. This process ensured the purity and accuracy of the selected T cells and B cells for further analysis.

Subsequently, T cell subsets were assigned based on the following criteria: central memory T cell (T_CM_) exhibited high expression of *SELL* and *CCR7*, while effector memory T cell expressed low *SELL* and *CCR7*; expression of *PDCD1, CTLA4, LAG3* and *ENTPD1* for CD8^+^ exhausted T cell; expression of *GZMA* CD4^+^ CTL; expression of *FOXP3, IL2RA* for regulatory T cell; expression of *TRBV20-1* for mucosal associated invariant T (MAIT) cell expressed; low expression of *PDCD1, CTLA4, LAG3* but high expression of *GZMB* for CD8^+^ CTL; expression of *MKI67, CCNA2* and *CCNB2* for proliferating T cell.

B cell subsets were assigned using the following criteria: high expression of *IGHD* and negative expression of *CD27* for naïve B cell; expression of *CD27*, negative expression of *IGHD* and low expression of *CCR7* and *CD40* for memory B cell; high expression of *CCR7* and *CD40* for activated B cell; negative expression of *CD27* and *IGHD* for double negative (DN) B cell; positive expression of *BCL6, AICDA, MEF2B* and *STMN1* for germinal center (GC) B cell; high expression of *IGHG1* and *IGHG3* for IgG ASC; high expression of *IGHA1* and *IGHA2* for IgA ASC.

### 2.5 TCR/BCR repertoire analysis

The TCR and BCR repertoire data generated from the 10x Genomics Cell Ranger pipeline were processed utilizing the scRepertoire v1.7.2 package (15). The information of barcode, TCR/BCR V(D)J and C germline gene usage, amino acid and nucleotide sequence of complementarity determining region (CDR) 3 was employed for downstream analysis. T cells or B cells with identical amino acid sequence in the CDR3 region were regarded as the same clonotype. Subsequently, the counts of identical expanded clonotypes representing clonal expansion among these clonotypes were computed and divided into six levels for T cells: Single: only one count per clonotype; Small: no more than three counts per clonotype; Medium: no more than 15 counts per clonotype; Large: no more than 50 counts per clonotype; Hyperexpanded: more than 50 counts per clonotype; NA: αβ TCR sequence was undetected, and six levels for B cells: Single: only one count per clonotype; Small: no more than three counts but more than one count per clonotype; Medium: no more than five counts but more than three counts per clonotype; Large: no more than ten counts but more than five counts per clonotype; Hyperexpanded: more than ten counts per clonotype; NA: BCR sequence was undetected. These different levels of counts of identical expanded clonotypes were visualized using the coordinates of UMAP derived from gene expression analysis conducted above.

Chord diagrams were employed to visualize the TCR interconnectivity among the distinct subsets. The counts of shared clonotypes between distinct CD4^+^ and/or CD8^+^ T cell subsets assigned in the gene expression analysis and unique clonotypes of these distinct T cell subsets were computed to construct a matrix for visualization. This visualization using chord diagrams was conducted using the circlize v0.4.15 package (16), where a chord connecting two different subsets represented shared clonotypes and the thicker these chords were, the greater counts of shared clonotypes the two subsets had. The counts of these unique clonotypes grouped by different CD4^+^ T cells, CD8^+^ T cells and B cells were also visualized using bar plots.

The diversity of these distinct subsets in CD4^+^ T cells, CD8^+^ T cells and B cells was estimated by five metrics: Shannon, inverse Simpson, Chao1 and inverse Pielou’s measure of species evenness (17–20). The Shannon Diversity Index was calculated using the following process: 1) count the total number of clonotypes (N), and the total number of TCR/BCR sequences in each clonotype (n). 2) calculate the proportion of each clonotype (pi) in the community via dividing n by N. 3) calculate the Shannon Diversity Index using the formula: -sum (pi * ln(pi)). The inverse Simpson Index was calculated using the formula: 1/sum (pi^2) and the Pielou Index was calculated by dividing the Shannon Diversity Index by the natural logarithm of the total clonotypes count. The Chao1 Index was calculated using the following process: 1) count the total number of clonotypes (N). 2) determine the number of clonotypes with single (n1) or double (n2) TCR/BCR sequence. 3) calculate the Chao1 Index using the formula: N + [n1(n1 - 1)]/[2 * (n2 + 1)].

In addition, MiXCR v4.0 (21) was used to calculate the frequency of somatic hypermutations (SHMs). An alignment-guided consensus algorithm was employed to reconstruct the BCR amino acid sequences and identify SHMs from the input of BCR nucleotide sequences. Subsequently, each mutation flagged by the algorithm employed to calculate the SHM frequency via dividing the mutation amino acid by the total number of amino acids. The output was visualized utilizing the Platypus v3.4.0 package (22) and was grouped by distinct B cell subsets assigned in gene expression analysis. The Pairwise Wilcoxon test was employed to calculate the p-values between the DN3 subset and the IgG ASC subset, as well as between the DN3 subset and the IgA subset, and the p-values were adjusted using the Benjamini-Hochberg method.

### 2.6 Single-cell trajectory analysis and RNA velocity analysis

The single-cell trajectory analysis in this study was conducted using Monocle3 v1.3.1 package (23). The two Seurat objects with barcodes, features and gene expression matrix, which contained conventional CD4^+^ T cells and CD8^+^ T cells, respectively, were transformed into objects suitable for Monocle3. Subsequently, these subsets information and UMAP coordinates from the gene expression analysis above were assigned to the objects. The single-cell trajectory analysis was carried out following these steps: 1) Graph learning: trajectory graphs for the whole conventional CD4^+^ T cells and CD8^+^ T cells were fit using Monocle3’s ‘learn_graph’ function, respectively. 2) Cell ordering in pseudotime: To arrange the cells along the trajectories, ‘order_cells’ function was utilized and the T_CM_ subset assigned in both conventional CD4^+^ T cells and CD8^+^ T cells was designated as the root of the trajectory. 3) Graph visualization: These T cells were plotted using the UMAP coordinates calculated in the gene expression analysis and colored by pseudotime. Finally, gene dynamic plots were produced using the ggplot2 v3.4.1 package, the average gene expression of specific genes in cells with identical pseudotime was calculated and these dot plots were visualized by the pseudotime order of these cells and colored by distinct subsets.

The RNA velocity analysis was performed using velocyto v0.17 and velocyto.R v0.6 package (24). The alignment bam files and barcode information of the three OSCC samples generated from 10x cellranger software were used as input data for velocyto, which is a command line tool. Subsequently, velocyto was used to classify the reads in bam files into distinct categories to yield loom files for the three OSCC samples and gene annotation gtf file and repeat masked gtf file from 10x GRCH38 Cell Ranger reference package was employed at this step. The output loom files contained matrices of spliced, unspliced and ambiguous reads of detected genes and were used for further analysis using velocyto.R v0.6 package in R 4.2.0 environment. The three loom files were merged firstly and divided into two objects with conventional CD4^+^ T cells and CD8^+^ T cells, respectively. The subset information and UMAP coordinates from the gene expression analysis above were assigned to the velocyto objects and RNA velocity estimation was performed for the two objects using ‘RunVelocity’ function from velocyto.R v0.6 package grouped by distinct conventional CD4^+^ T cell and CD8^+^ T cell subsets. Following this, subsequent visualization based on the UMAP coordinates generated in the gene expression analysis was produced using ‘show.velocity.on.embedding.cor’ function in velocyto.R v0.6 package.

### 2.7 Hierarchical clustering analysis and cell-cell communication analysis

The average gene expression matrix of each T cell subset assigned in gene expression analysis was calculated for the hierarchical clustering analysis. The hierarchical clustering analysis was executed using ‘hclust’ function and the algorithm ward.D2 in R 4.2.0 environment (25). The hierarchical clustering analysis results were visualized in a dendrogram to elucidate the relationships among the subsets.

The cell-cell communication analysis of cellular crosstalk between B cell and conventional CD4^+^ T cell subsets was carried out using CellphoneDB v4 (26). Gene expression data, along with cell subset annotation information for these T cells and B cells from the gene expression analysis generated above were exported for the prediction of enriched receptor-ligand interactions. The interaction was predicted on the expression of a receptor by one cell type and a ligand by another cell type using cellphonedb v4.1.0 package in the python 3.10.9 environment. The generated output files containing p-values indicating the statistical significance of specific interaction pairs between conventional CD4^+^ T cells and B cells and the average expression level of each interaction pair between each cell pair were utilized for subsequent visualization in the R 4.2.0 environment. The quantity of significant receptor-ligand interactions between conventional CD4^+^ T cells and B cells was computed and visualized in a heatmap. The specific receptor-ligand pairs were depicted in dot plots, where dot size indicated the negative logarithm base 10 of p-values and gradient color indicated the logarithm base 2 of the average expression of each interaction pair. Both visualizations were produced using the ggplot2 v3.4.1 package.

### 2.8 High dimensional weighted gene co-expression network analysis and gene ontology enrichment analysis

All the T cell subsets identified in the gene expression analysis were subjected to WGCNA at a single-cell level using the hdWGCNA v0.2.18 package (27). To optimize the T cell dataset for the hdWGCNA pipeline, metacells were constructed for each distinct T cell subset, and the harmony v0.1.1 package (28) was employed for the dimensionality reduction. Then k-Nearest Neighbors algorithm was performed to identify groups of similar T cells for aggregation. The resulting metacells gene expression matrix was subsequently used in the WGCNA. The metacell gene expression matrix from the CD4^+^CTL subset was specified for the co-expression network analysis and the different co-expression modules resulting were visualized in a dendrogram.

Following this, the harmonized module eigengenes (hMEs) were computed across the entire T cell dataset based on the co-expression modules obtained from the WGCNA and were visualized in conjunction with the UMAP calculated in the Seurat object using a feature plot. The hME of module 3 (M3) in the CD4^+^CTL subset was further visualized in T cell subsets using a violin plot. Genes in each co-expression module were assessed based on the eigengene-based connectivity, or kME, and were subsequently visualized in each module arranged according to kME. The genes with top 10 kME values were displayed in this plot.

The genes with top 100 highest kME values in M3 were extracted for GO enrichment analysis (29), which was also conducted in the R 4.2.0 environment. The gene symbols were converted to Entrez IDs using the stringr v1.5.0 package. Following this, the GO enrichment analysis was performed using the clusterProfiler v4.7.1.002 package (30). The top 10 biological processes (BPs) were displayed in a dot plot.

### 2.9 Single-cell regulatory network inference and clustering analysis

The subset information and gene expression matrix of all T cells generated by Seurat was used for SCENIC analysis using the SCENIC v1.3.1 package in the R 4.2.0 environment (31). SCENIC settings were initialized using the following motif database: hg19-500bp-upstream-7species.mc9nr.feather and hg19-tss-centered-10kb-7species.mc9nr.feather. Subsequently, a soft gene filter was applied to remove the genes in very few cells (less than 1% cells) and the transcription factor (TF) list and gene expression matrix were exported to text format to run GRNboost. Co-expression modules with TFs were computed based on the prepared matrix using the GRNboost v0.1.5 package in the python 3.10.9 environment and the output was further analyzed in the R 4.2.0 environment. The RcisTarget v1.18.2 package was employed to analyze TF motif for the identification of regulons. Then the regulon activity in the T cells was scored using the AUCell v1.20.2 package. The average regulon activity as well as these identified regulons across different T cell subsets was visualized in a heatmap.

### 2.10 Hematoxylin and eosin staining

The formalin-fixed, paraffin embedded (FFPE) OSCC tissue sections, with a thickness of 4 μm, were subjected to a deparaffinization process in xylene for 10 min, followed by a rehydration procedure through a graded series of ethanol solutions with decreasing concentrations: 100% ethanol for 10 min, 90% ethanol for 5 min, 80% ethanol for 5 min, and 70% ethanol for 5 min. Subsequently, the slides were rinsed in water for 2 min before being immersed in hematoxylin for 1 min to stain the nuclei. After thorough washing, the washed slides were stained with eosin for 5 min, and afterwards dehydrated in a gradient series of ethanol solutions and xylene. Finally, the slides were mounted with the coverslip and acrylic resin dissolved in xylene. An Olympus IX83 inverted microscope was employed to observe the prepared slides and capture images.

### 2.11 Multicolor immunofluorescence staining and cell quantification

All tissue samples for HE and IF staining were fixed in neutral buffered formalin for less than 72 hours. Those formalin-fixed paraffin-embedded (FFPE) blocks were placed in a room temperature, dark, low humidity environment until use. Although paraffin blocks can be stored for 25 years for immunohistochemistry and ten years for protein-requiring platforms, samples stored for less than ten years were used in this study (32). IF staining was conducted immediately after the FFPE blocks were cut into sections to avoid the quick antigenicity decay of samples (33, 34). FFPE OSCC tissue sections were subjected to deparaffinization and rehydration. The sections were washed with Tris-buffered saline with tween 20 (TBST) three times for 3 min each wash. Antigen retrieval was carried out using either AR6 buffer (AR6001KT, Akoya) or AR9 buffer (AR9001KT, Akoya), depending on the specific primary antibodies required. The information and condition of primary antibodies were provided in **Supplementary Table 2**. This process involved microwave treatment (MWT) for 15 min, followed by cooling to RT. After the MWT step, the sections were washed in TBST with agitation three times for 2 min each wash. The sections were then blocked by applying an antibody diluent/block buffer (ARD1001EA, Akoya) for 10 min at RT.

Primary antibodies were diluted in diluent/block buffer (ARD1001EA, Akoya) and subsequently applied to the sections for incubation in a humid chamber. Following the primary antibody incubation, the sections were washed three times for 3 min each wash and incubated with an Opal Polymer HRP Ms + Rb secondary antibody (ARH1001EA, Akoya). Subsequently, the sections were washed three times for 3 min each wash for signal amplification using TSA Amplification Reagent. The TSA Amplification Reagent used in this study included TSA Plus Fluorescein (NEL741001KT, Akoya), TSA Plus Cyanine 3 (NEL744001KT, Akoya), TSA Plus Cyanine 5 (NEL745001KT, Akoya, and Opal 780 Reagent Pack (FP1501001KT, Akoya). Following signal amplification, MWT was performed for multiplexing. When all target proteins were detected, the sections were stained with Spectral DAPI (FP1490, Akoya) for 5 min at RT and subsequently mounted using coverslips with VECTASHIELD PLUS Antifade Mounting Medium (H-1900, Vector Laboratories).

An Olympus IX83 inverted microscope was used to examine the prepared sections and capture images. The captured images were used to quantify cells in StrataQuest 7.0 software (TissueGnostics). Positivity or negativity of markers was determined by the cutoff values of intensity and area parameters established by experienced pathologists. Lymphocyte subsets were identified by the following criteria: CD4^+^ Granzyme A^+^ for CD4^+^CTLs; CD4^+^ T-bet^+^ for T helper 1 (Th1) cells; CD4^+^ GATA3^+^ for T helper 2 (Th2) cells; CD4^+^ RORγT^+^ for T helper 17 (Th17) cells; CD4^+^ FoxP3^+^ for Tregs; CD4^+^ ICOS^+^ CXCR5^+^ for T follicular helper (Tfh) cells; CD4^+^ CCR7^+^ CD45RA^-^ for CD4^+^ T_CM_; CD8^+^ Granzyme A^+^/Granzyme B^+^ for CD8^+^ CTL; CD8^+^ PDCD1^+^/CTLA4^+^ CD39^+^ for CD8^+^ T_EX_; CD19^+^ IgD^-^ CD27^-^ for DN B cells; CD19^+^ IgD^-^ CD27^-^ CXCR5^+^ CD11c^-^ for DN1 B cells; CD19^+^ IgD^-^ CD27^-^ CXCR5^-^ CD11c^+^ for DN2 B cells; CD19^+^ IgD^-^ CD27^-^ CXCR5^-^ CD11c^-^ for DN3 B cells; CD19^+^ IgD^-^ CD27^-^ CXCR5^-^ CD11c^-^ for DN4 B cells.

### 2.12 Statistical analysis

Statistical analysis and data visualization were performed in the R 4.2.0 environment. The Kaplan-Meier (K-M) curve was plotted, and survival analysis was executed by comparing two Kaplan-Meier curves through following steps (35): 1) Create a survival data table: A progression of OSCC, which was recurrence or metastasis here, was identified as the event of interest. The 20 patients (**Supplementary Table 1**, patients marked as ‘multi-color IF, K-M and COX analysis’ in the Application column) with progression-free duration, measured in days, was used as the elapsed time to the event of interest and one of the cases was not followed up for the full five years and was therefore considered as censored data in the current study. 2) Separate into two categories, high and low density, based on the density of CD4^+^ CTLs: A logistic regression model was fit in this step for the event of interest and the density of CD4^+^ CTLs. Receiver operating characteristic and area under the curve values were calculated to determine the optimal cutoff point. The cutoff value of the CD4^+^ CTL density was calculated using the formula: log (BCP/(1 – BCP) – β_0_)/β_1_, where β_0_ is the intercept and β_1_ is the coefficient of the CD4^+^ CTL density, and BCP is the best cut off point from the logistic regression model. 3) Draw K-M curves: The two K-M curves, grouped by high and low CD4^+^ CTL density, were plotted using the survimer v0.4.9 package in R 4.2.0 environment. 4) Compare the two categories: The log-rank test was used to estimate the statistical significance between the two categories. A p-value less than 0.05 was considered statistically significant.

Survival analysis utilizing Cox proportional hazards regression was performed on event of interest, which was progression of OSCC as above, and variables, as well as covariables, from the 20 patients same as above (**Supplementary Table 1**, patients marked as ‘multi-color IF, K-M and COX analysis’ in the Application column) in R 4.2.0 environment through the following steps (36): 1) Filter variables: Cox proportional hazards models were constructed for the following variable, respectively: a) density of CD4^+^ CTL, Treg, CD4^+^ T_CM_, Th1, Th2, Th17 and Tfh subsets, b) age, c) sex, d) T stage, e) N stage, f) differentiation of carcinoma. Variables exhibiting significance were selected for next step, where a p-value less than 0.05 was considered statistically significant. 2) Construction of Cox models: Cox models including CD4^+^ CTL density and N classification, Treg density and N classification, Th2 density and N classification were constructed, respectively. 3) Significance test for variables: The partial likelihood ratio test was used to calculate p-values for these variables in these distinct models, and a p-value of less than 0.05 was considered statistically significant.

## 3 Results

### 3.1 Single-cell RNA sequencing reveals the landscape of infiltrated immune cells in OSCC tissues

**Figure 1:**
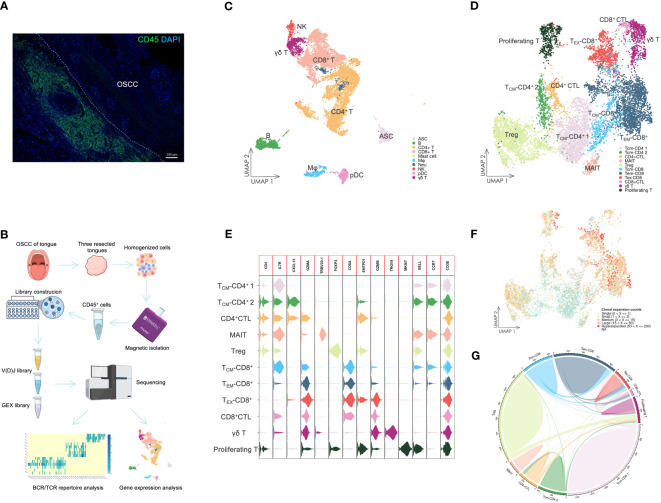
The landscape of infiltrating immune cells. **(A)** IF staining of CD45^+^ cells in OSCC tissue. The staining showed that dense immune cells had infiltrated in OSCC of tongue tissue. Scale bars: 100 µm. **(B)** Overview of scRNA-seq. Resected tongues from three patients with OSCC underwent homogenization, from which CD45^+^ cells were extracted by magnetic isolation. GEX and V(D)J libraries were subsequently constructed using these cells for sequencing, and the sequencing data were analyzed by various methods. **(C)** UMAP visualization of infiltrating immune cells with assigned clusters. This displayed a variety of immune cells in OSCC tissues. ASC: antibody-secreting cell; B: B cell; CD4^+^ T: CD4^+^ T cell; CD8^+^: CD8^+^ T cell; γδ T: γδ T cell; Mφ: macrophage; Neu: neutrophil; NK: natural killer cell; pDC: plasmacytoid dendritic cell. **(D)** UMAP visualization of T cells colored by assigned T cell subsets. Various T cell subsets were identified by unsupervised clustering and assigned based on expression of specific marker genes. T_CM_-CD4: CD4^+^ central memory T cell; CD4^+^ CTL: CD4^+^ cytotoxic T lymphocyte; MAIT: mucosal associated invariant T cell; Treg: regulatory T cell; T_CM_-CD8: CD8^+^ central memory T cell; T_EM_-CD8: CD8^+^ effector memory T cell; T_EX_-CD8: CD8^+^ exhausted T cell; γδ T: γδ T cell; Proliferating T: proliferating T cell. **(E)** Violin plot of gene expression in T cell subsets. This plot showed the marker gene expression used to assign T cell subsets. **(F)** UMAP visualization of T cells combined with TCR clonal expansion. The specific gradient color denotes different degree of clonal expansion in TCRs. NA: αβ TCR sequence was undetected. **(G)** Chord diagrams of the unique and shared TCR clonotype counts in T cell subsets. The chords connecting two different subsets represent shared TCR clonotypes.

To investigate infiltrated immune cells in OSCC tissues, we homogenized tongue samples from three patients with OSCC and isolated CD45^+^ cells that had infiltrated into the resected tongues (**Figure 1A**). We then analyzed the CD45^+^ cells by single-cell RNA sequencing (scRNA-seq) and TCR/BCR repertoire sequencing (**Figure 1B**).

The scRNA-seq data were visualized using UMAP dimensionality reduction (**Figure 1C**, **Supplementary Figure 1**). We then assigned immune cell types to distinct clusters based on leukocyte phenotype genes (**Supplementary Figures 2, 3**). The types of infiltrated immune cells identified included T cells, B cells, ASCs, natural killer cells, macrophages, pDCs, neutrophils, and mast cells.

We extracted the T cells and performed further cell clustering analysis (**Figure 1D**). Subsequently, we assigned T cell subset types to the clusters based on marker genes (**Figure 1E**, **Supplementary Figure 3**). A CD4^+^ T cell subset expressing *GZMA* was designated as the CD4^+^ CTL subset. The subset labeled T_CM_-CD4^+^ 2 exhibited high expression of *CXCL13*, which distinguished it from the T_CM_-CD4^+^ 1 subset. The MAIT subset was identified by expression of *TRBV20-1* (37). The Treg subset expressed a high level of *FOXP3*, whereas only the γδ T cell subset expressed *TRGV9*. T_CM_-CD8^+^ and T_EM_-CD8^+^ subsets were assigned based on the expression levels of *SELL* and *CCR7*. Both CD8^+^ CTL and T_EX_-CD8^+^ subsets displayed elevated expression of *GZMB*. However, the T_EX_-CD8^+^ subset also expressed *ENTPD1* and other IR genes. The proliferating T cell subset represented T cells undergoing proliferation and included both CD4^+^ and CD8^+^ T cells (**Figure 1E**).

The different levels of TCR sequence counts of each clonotype in T cells were also visualized through UMAP (**Figure 1F**). Highly expanded clonotypes were predominantly found in CD4^+^ CTL, Treg, T_EM_-CD8^+^, and T_EX_-CD8^+^ T cell subsets. To circumvent potential misinterpretation of novel subsets due to doublets generated during library construction, we further investigated the shared TCR sequences among T cell subsets (**Figure 1G**). The CD4^+^ CTL subset shared TCR sequences with T_CM_-CD4^+^ 2 and proliferating T cell subsets, but did not share TCR sequences with either Treg or CD8^+^ T cell subsets. This revealed that CD4^+^ CTLs were a unique subset of T cells and related to other conventional CD4^+^ T cell subsets, such as the T_CM_-CD4^+^ 2 subset.

### 3.2 CD4^+^ CTLs represent a unique subset and may differentiate from other CD4^+^ T cells

**Figure 2:**
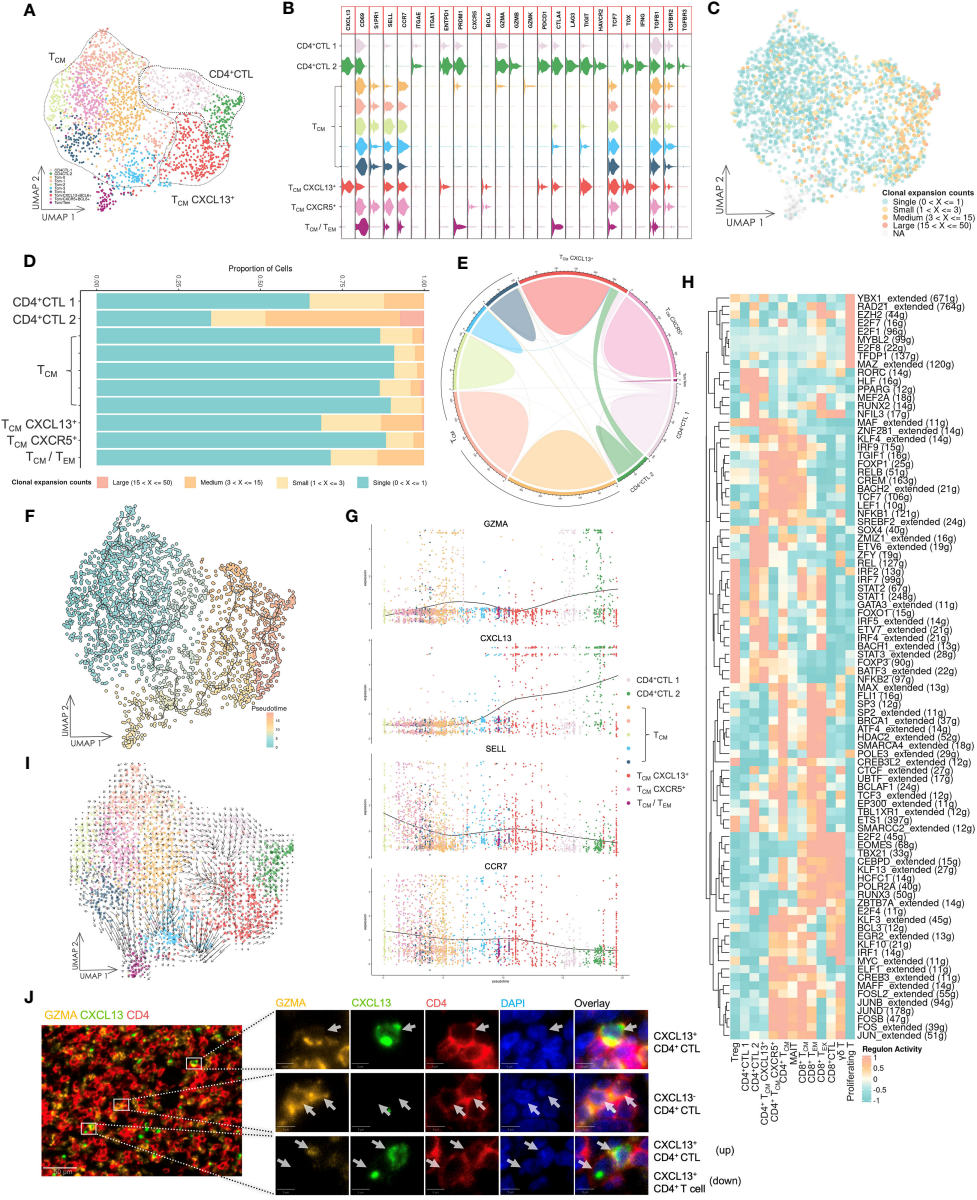
Gene expression and TCR repertoire analysis of conventional CD4^+^ T cells. **(A)** UMAP visualization of conventional CD4^+^ T cells. CD4^+^ CTL: CD4^+^ cytotoxic T lymphocyte; T_CM_: central memory T cell; T_CM_ CXCL13^+^: *CXCL13*-expressing central memory T cell; T_CM_ CXCR5^+^: *CXCR5*-expressing central memory T cell; T_CM_/T_EM_: central memory T cell and effector memory T cell. **(B)** Violin plot of gene expression in CD4^+^ T cell subsets. The plot depicts expression of genes associated with T cell functions in distinct subsets. **(C)** UMAP visualization of conventional CD4^+^ T cells integrated with TCR clonal expansion. The gradient color represents different degree of clonal expansion in distinct TCR clonotypes. NA: αβ TCR sequence was undetected. **(D)** Proportional stacked bar plot of clonal expansion in conventional CD4^+^ T cell subsets. A high level of clonal expansion was predominantly found in the CD4^+^ CTL subsets. **(E)** Chord diagrams of unique and shared TCR clonotype counts in conventional CD4^+^ T cell subsets. The chords connecting two different subsets represent shared TCR clonotypes. **(F)** UMAP visualization of conventional CD4^+^ T cells colored by pseudotime and integrated with single cell trajectories. The T_CM_ subset was set as the root node of single cell trajectories. **(G)** Gene dynamic plot based on pseudotime. Cells were ordered in accordance with pseudotime, and the average expression of these genes in cells with identical pseudotime was calculated and visualized in this plot. **(H)** Heat map of the top 10 active regulons in T cell subsets. An extended regulon refers to a regulon that has been expanded to include both direct and indirect targets of a regulon. The counts of these genes are indicated by parenthesis following the regulons. **(I)** UMAP visualization integrated with RNA velocity analysis of conventional CD4^+^ T cells. The direction of the arrow represents the predicted direction of change in cell state, and the length of the arrow represents the predicted speed of change in cell state. **(J)** Multicolor IF staining of CD4^+^ CTLs and CXCL13^+^ CD4^+^ T cells in OSCC tissue. Scale bars: 50 µm (low magnification) and 5 µm (high magnification).

To investigate the association between CD4^+^ CTLs and other conventional CD4^+^ T cells, we analyzed CD4^+^ CTL, T_CM_-CD4^+^ 1, and T_CM_-CD4^+^ 2 subsets. Additionally, we renamed some subsets using marker genes from the clustering analysis, such as T_CM_-CXCL13^+^ and T_CM_-CXCR5^+^ subsets (**Figures 2A, B**, **Supplementary Figure 4**). Both CD4^+^CTL 1 and CD4^+^CTL 2 subsets expressed cytotoxic gene *GZMA*. However, the CD4^+^CTL 2 subset also expressed *GZMB* and other IR genes expressed in exhausted CD8^+^ T cells, such as *PDCD1*, *CTLA4*, *LAG3*, *TIGIT*, *HAVCR2*, and *TOX* (38). Notably, T cells in CD4^+^ CTL and T_CM_-CXCL13^+^ subsets exhibited high clonal expansion, low diversity and low frequency of unique clonotypes, indicating that these subsets had experienced activation and proliferation because of antigen specificity (**Figures 2C, D**, **Supplementary Figure 5**). Conversely, the T_CM_ subset exhibited low TCR clonal expansion, suggesting a lack of antigen specificity.

The CD4^+^ CTL subset had shared TCR sequences with the T_CM_-CXCL13^+^ subset (**Figure 2E**), revealing a potential developmental relationship between these two subsets. Consequently, we performed pseudotime analysis based on calculations and predictions for estimating single cell trajectories, although it would be limited in accuracy or confidence (**Figure 2F**). By integrating this analysis with the TCR repertoire analysis, our findings demonstrated that the CD4^+^ CTL subset was the most terminally developed subset among all CD4^+^ T cell subsets. Furthermore, the T_CM_-CXCL13^+^ subset represented an activated subset that had been presented antigens by antigen-presenting cells and potentially differentiated into the CD4^+^ CTL subset upon continuous exposure to antigens from cancer cells. A gene dynamic plot revealed alterations in expression of certain genes (**Figure 2G**, **Supplementary Figure 6**). During pseudotime progression from T_CM_ to CD4^+^ CTLs, *GZMA* and *CXCL13* exhibited continuous upregulation, while *CCR7* and *SELL* displayed downregulation. This suggested that activated T_CM_ cells were predisposed to residing in peripheral tissue and acquired the ability to respond to cancer cells directly or indirectly.

Because differentiation of T cells is predominantly driven by specific gene regulatory networks that are primarily mediated by transcription factors (TFs) and cofactors, we employed SCENIC, a computational tool designed to infer regulatory information from scRNA-seq data, to predict potential TFs and gene regulatory networks in these T cell subsets (**Figure 2H**). Notably, the CD4^+^ CTL subset displayed a different group of regulons compared with other T cell subsets, separating them from other T cell subsets.Additionally, we carried out RNA velocity analysis to estimate the cell state in various subsets (**Figure 2I**). The length of the arrow was longer in T_CM_-CXCL13^+^ and some T_CM_ subsets compared with CD4^+^ CTL and other T_CM_ subsets, indicating that the cell state in the T_CM_-CXCL13^+^ subset was changing at a fast rate, whereas CD4^+^ CTLs may have halted their development and maintained a stable cell state. Multicolor IF staining in tissue lesions confirmed the CD4^+^ CTL subset and their aggregation by the expression of CD4, GZMA and CXCL13 (**Figure 2J**).

### 3.3 CD4^+^ CTLs exhibit cytotoxicity and may induce apoptosis of tumor cells

**Figure 3:**
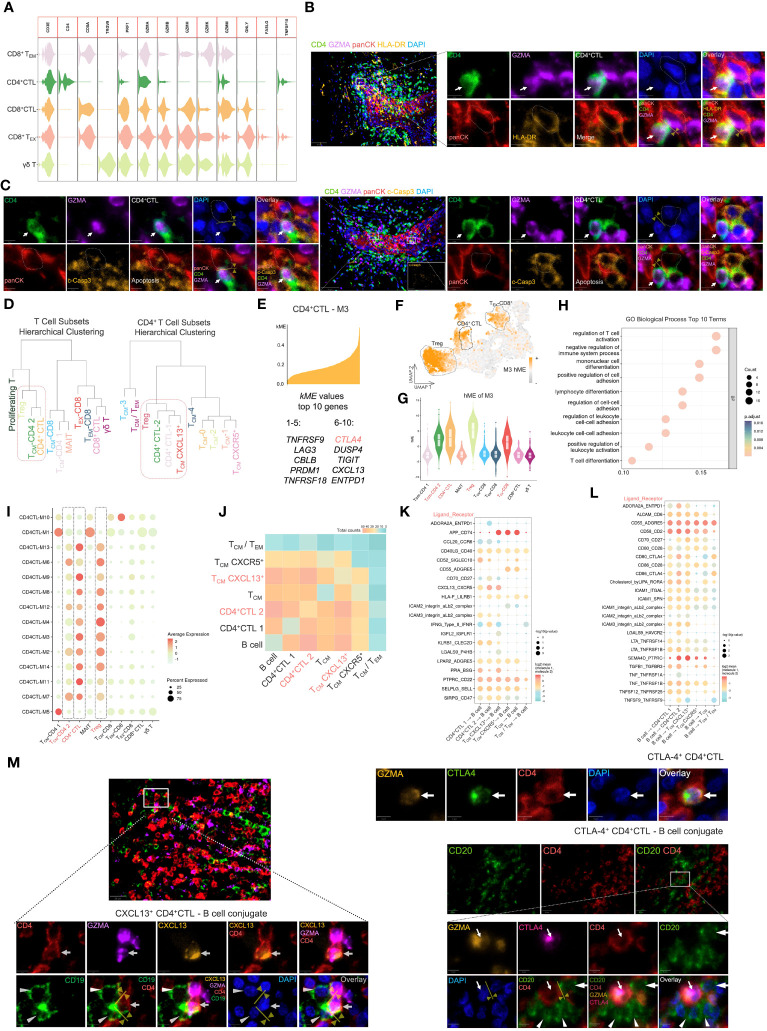
The CD4^+^ CTL subset may exert pleiotropic effects in tumor immunity. **(A)** Violin plot of gene expression related to cytotoxicity in several cytotoxic T cell subsets. **(B)** Multicolor IF staining of CD4^+^ CTLs and HLA-DR^+^ tumor cells. Conjugation between these two cell types is depicted by the yellow arrow and line. GZMA: granzyme A; panCK: pan-cytokeratin; c-Casp3: cleaved caspase-3. Scale bars: 50 µm (low magnification) and 5 µm (high magnification). **(C)** Multicolor IF staining of CD4^+^ CTLs and apoptotic tumor cells. The white arrow indicates CD4^+^ CTLs, and the dotted box indicates apoptotic tumor cells. Scale bars: 50 µm (low magnification) and 5 µm (high magnification). **(D)** Visualization of hierarchical clustering analysis in T cell subsets. **(E)** Visualization of genes in module 3 (M3). The top 10 kME value genes are displayed in this plot. **(F)** Feature plot of M3 hMEs. Expression of M3 hMEs was calculated in single cells and visualized on UMAP. **(G)** Violin plot of M3 hMEs. Expression of M3 hMEs was calculated in single cells and visualized in various T cell subsets. **(H)** Dot plot of the top 10 terms of biological processes in GO enrichment analysis. The enrichment p-values were calculated using the hypergeometric distribution test and adjusted by the Benjamini–Hochberg method. **(I)** Dot plot of hMEs in all modules. Expression of hMEs in all modules was calculated in single cells and visualized in various T cell subsets. The dotted box indicates the specific T cell subsets. **(J)** Heat map of significant receptor–ligand interactions. The gradient color represents the quantity of significant interactions. **(K-L)** Dot plot of specific receptor–ligand pairs. The permutation test was used to calculate the p-value. (**M)** Multicolor IF staining of CXCL-13-producing CD4^+^ CTLs (left), CTLA-4-expressing CD4^+^ CTLs (upper right), and conjugation with B cells (lower right). Conjugation between these cells is depicted by the yellow arrow and line. Scale bars: 20 µm (low magnification) and 5 µm (high magnification).

The potential anti-tumor capacity of CD4^+^ CTLs has been demonstrated across multiple tumor types (39). To investigate the anti-tumor activity of CD4^+^ CTLs in OSCC, we compared the expression level of cytotoxic genes between the CD4^+^ CTL subset and other cytotoxic T cell subsets (**Figure 3A**). Our results indicated that the CD4^+^ CTL subset expressed *PRF1*, *GZMA*, and *GZMB*, indicating that CD4^+^ CTLs may induce apoptosis in tumor cells via the release of cytotoxic mediators, especially granzyme B and perforin. The expression level of cytotoxic genes in the CD4^+^ CTL subset, such as *GZMA* and *GZMB*, was elevated compared with that in the other CD4^+^ T cell subsets (**Figure 2B**), but did not appear to reach comparable levels with those in the CD8^+^ T cell subset. An intriguing observation was that the CD4^+^ CTL subset also expressed *TNFSF10* that encodes the protein TRAIL. Considering that TRAIL and its corresponding receptor, TRAIL receptor, induce apoptosis in tumor cells, this suggests an alternative apoptotic pathway independent from granzyme B and perforin pathways.

Because CD4^+^ CTLs mediate cell death in an HLA class II-restricted manner (40), we examined the proximity of CD4^+^ CTLs and tumor cells expressing HLA-DR by multicolor IF staining (**Figure 3B**). The staining suggested that CD4^+^ CTLs were in contact with HLA-DR-positive tumor cells. We further observed the proximity of CD4^+^ CTLs and apoptotic tumor cells (**Figure 3C**). These data indicated the probable anti-tumor efficacy of CD4^+^ CTLs.

### 3.4 CD4^+^ CTLs and Tregs share similar functional co-expression modules

To elucidate the correlation between these T cell subsets, we calculated the average gene expression of distinct T cell subsets and conducted hierarchical clustering analysis (**Figure 3D**). The gene expression patterns of T_CM_-CD4 2 (i.e., T_CM_-CXCL13^+^) and CD4^+^ CTLs appeared to be similar, whereas all CD8^+^ T cell subsets merged into one cluster at an earlier stage. Notably, the CD4^+^CTL 2 subset displayed a gene expression pattern resembling that of the Treg subset.

To further investigate the specific similarities in the gene expression patterns between these subsets, we performed WGCNA, which is a powerful analytical tool designed to identify modules of highly correlated genes, on the CD4^+^ CTL subset at the single cell level (**Supplementary Figure 7**). An intriguing module of highly correlated genes, designated as M3, included IR genes, such as *CTLA4*, *LAG3*, *TIGIT*, *ENTPD1*, *TNFRSF9*, and *TNFRSF18* (**Figure 3E**), which were upregulated in CD4^+^ CTL, Treg, and T_EX_-CD8^+^ subsets (**Figures 3F, G**). Subsequently, we performed Gene Ontology (GO) biological process analysis on M3 (**Figure 3H**), which revealed that M3 was associated with terms like regulation of T cell activation and negative regulation of immune system processes. Additionally, we compared all identified modules across the various subsets (**Figure 3I**, **Supplementary Figure 8**) and found that hME expression of modules in CD4^+^ CTLs and Tregs was similar.

### 3.5 CD4^+^ CTLs interact with B cells and potentially elicit pleiotropic effects for tumor immunity

A crucial function of CD4^+^ T cells is mediating humoral immunity together with B cells. Consequently, we focused on the cell communication and interactions between the CD4^+^ T cell subset and B cells. Enriched ligand–receptor interactions between CD4^+^ T cell subsets and B cells were predicted by expression of a receptor in CD4^+^ T cell subsets and a ligand in B cells using CellphoneDB and vice versa. We then visualized the number of predicted significant interactions using a heat map (**Figure 3J**). CD4^+^CTL 2 and T_CM_-CXCL13^+^ subsets demonstrated particularly strong interactions with B cells, followed by the CD4^+^CTL 1 subset, indicating that both CD4^+^CTL and T_CM_-CXCL13^+^ subsets exhibited enhanced communication with B cells.

Next, we visualized the specific ligand–receptor interactions between CD4^+^ T cell subsets and B cells using a dot plot (**Figures 3K, L**). Upregulation of ligand–receptor pairs, such as CXCL13–CXCR5 and ICAM1,3–α_L_β_2_, between CD4^+^ CTL and B cell subsets or T_CM_-CXCL13^+^ and B cell subsets facilitated contact between these cells. Multicolor IF staining also demonstrated that CXCL13-producing CD4^+^ CTLs attracted B cells (**Figure 3M**).

The upregulation of CD40LG–CD40 and CD28–CD80/CD86 ligand–receptor interactions between CD4^+^ CTL and B cell subsets or T_CM_-CXCL13^+^ and B cell subsets represented promotion of activation and further development of T and B cells. Interestingly, we observed a strong IFNG–IFNR interaction between CD4^+^CTL 2 and B cell subsets, and *IFNG* was upregulated in the CD4^+^CTL 2 subset (**Figure 2B**). IFN-γ promotes ASC development (41), and signaling from IFN- γ has been implicated in promoting germinal centers (42). This suggested that the CD4^+^CTL 2 subset was associated with B cell differentiation. Furthermore, secreted IFN-γ recruits monocytes to the tumor site and activates them (4), resulting in a positive effect on tumor immunity.

Conversely, the upregulation of certain ligand–receptor interactions, such as ADORA2A–ENTPD1, CLEC2D–KLRB1, CD80–CTLA4, and CD86-CTLA4, is indicative of inhibitory regulation of T and B cells (43–46), implying a detrimental effect on tumor immunity. We further investigated the typical IR molecule CTLA-4 by multicolor IF (**Figure 3M**). The results showed that CTLA-4-expressing CD4^+^ CTLs attracted and conjugated with CD20^+^ B cells, suggesting potential suppression of B cell responses (47).

### 3.6 Differential gene expression patterns in tumor-infiltrating CD8^+^ T cells

**Figure 4:**
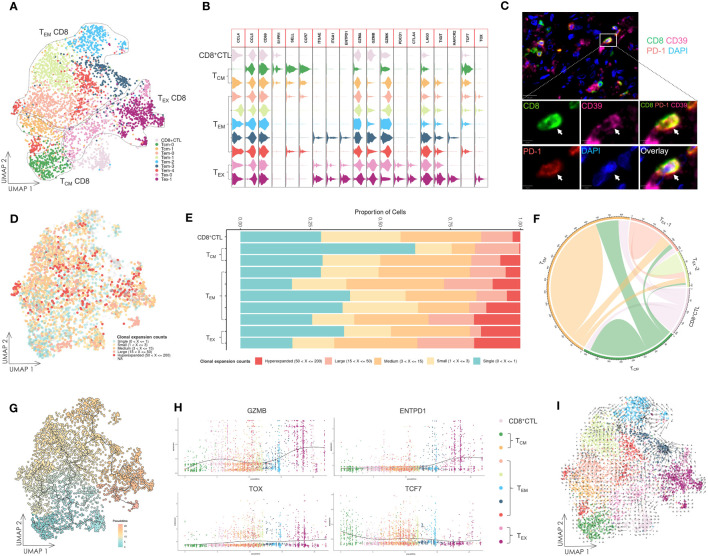
Gene expression and TCR repertoire analysis of CD8^+^ T cells. **(A)** UMAP visualization of CD8^+^ T cells. T_CM_ CD8: central memory CD8^+^ T cell; T_EM_ CD8: effector memory CD8^+^ T cell; T_EX_ CD8: exhausted CD8^+^ T cell; CD8^+^ CTL: CD8^+^ cytotoxic T lymphocyte. **(B)** Violin plot of gene expression in CD8^+^ T cell subsets. **(C)** Multicolor IF staining of CD8^+^ exhausted T cells in OSCC tissue samples. The white arrow indicates CD39^+^ PD-1^+^ CD8^+^ exhausted T cells. Scale bars: 20 µm (low magnification) and 5 µm (high magnification). **(D)** UMAP visualization of CD8^+^ T cells integrated with TCR clonal expansion. The gradient color represents the different degree of clonal expansion in distinct TCR clonotypes. NA: αβ TCR sequence was undetected. **(E)** Proportional stacked bar plot of clonal expansion in CD8^+^ T cell subsets. **(F)** Chord diagrams of unique and shared TCR clonotype counts in CD8^+^ T cell subsets. The chords connecting two different subsets represent shared TCR clonotypes. **(G)** UMAP visualization of CD8^+^ T cells colored by pseudotime and integrated with single cell trajectories. **(H)** Gene dynamic plot based on pseudotime. Cells were ordered in accordance with the pseudotime, and the average expression of these genes in cells with identical pseudotime was calculated and visualized in this plot. **(I)** UMAP visualization integrated with RNA velocity analysis of CD8^+^ T cells. The direction of the arrow represents the predicted direction of the change in cell state, and the length of the arrow represents the predicted speed of change in cell state.

The subset of CD8^+^ T cells was primarily determined by expression of marker genes, such as *SELL*, *CCR7*, *ENTPD1*, *GZMB*, *PDCD1*, *CTLA4*, and *LAG3* (**Figures 4A, B**, **Supplementary Figure 9**). The T_CM_ subset was characterized by high expression of *SELL* and *CCR7*, and low expression of *GZMB*. Conversely, T_EM_, CD8^+^ CTL and T_EX_ subsets exhibited low or negative expression of *SELL* and *CCR7*. *GZMB* was highly expressed in one cluster in the T_EM_ subset and in CD8^+^ CTL and T_EX_ subsets. The T_EX_ subset further demonstrated high expression of IR genes, such as *PDCD1*, *CTLA4*, *LAG3*, and *TIGIT*. A cluster of CD8^+^ T cells in the T_EX_ subset had increased expression of *ENTPD1* encoding CD39, *HAVCR2* encoding TIM-3, and *TOX*, which indicated advanced exhaustion, suggesting a late dysfunctional state of these cells (38). Multicolor IF staining displayed the exhausted CD8^+^ T cells in OSCC tissue samples (**Figure 4C**).

To investigate the interrelationship among these T cell subsets, TCR repertoire analysis was conducted and visualized (**Figures 4D, E**, **Supplementary Figure 10**). Except for the T_CM_ subset, we found that other T cell subsets exhibited high clonal expansion in clonotypes, and the most oligoclonally expanded cells, low TCR diversity and low frequency of TCR unique clonotypes were observed in the T_EX_ subset. Many shared TCR sequences were observed among the subsets (**Figure 4F**), suggesting that T cells with the same clonotype existed in different cell states.

### 3.7 Tumor-specific CD8^+^ T cells exhibit consistent differentiation to dysfunctional states

We performed single cell trajectory analysis of these CD8^+^ T cells, and the T_CM_ subset was designated as the root of the trajectory to calculate pseudotime (**Figure 4G**). Together with the analysis of shared TCR sequences, it revealed that certain tumor-specific cells in the T_CM_ subset had developed into T_EM_ cells and ultimately to T_EX_ cells. This suggested persistent differentiation towards dysfunctional states during tumorigenesis.

Next, we tracked several genes using a gene dynamic plot (**Figure 4H**, **Supplementary Figure 11**), and found that *GZMA* and *GZMB* expression was continuously upregulated during differentiation. Similarly, genes encoding IRs, including *ENTPD1*, *PDCD1*, *CTLA4*, *LAG3*, and *HAVCR2*, showed a pattern of upregulated expression. Furthermore, *TOX* and *TCF7*, which encode two critical TFs, TOX and TCF1, during T cell exhaustion (48), demonstrated an inverse expression pattern characterized by a decrease in *TCF7* expression and an increase in *TOX* expression. Additionally, downregulated expression of *SELL* encoding the integrin CD62L and *CCR7*, alongside upregulated expression of *ITGA1* and *ITGAE* encoding CD49a and CD103, respectively, indicated tissue-resident development during differentiation.

Further insights into the cell state were achieved by RNA velocity analysis of CD8^+^ T cells (**Figure 4I**). Notably, the arrow length, which is indicative of the rate of the cell state change, was longer in several T_EM_ subsets compared with T_EX_ subsets. This implies a faster rate of cell state change in T_EM_ subsets. Conversely, T_EX_ subsets demonstrated relative stability in their cell state, suggesting that these cells had halted their development and maintained a consistent cell state.

### 3.8 Various subsets of B cells and ASCs infiltrate into OSCC lesions

**Figure 5:**
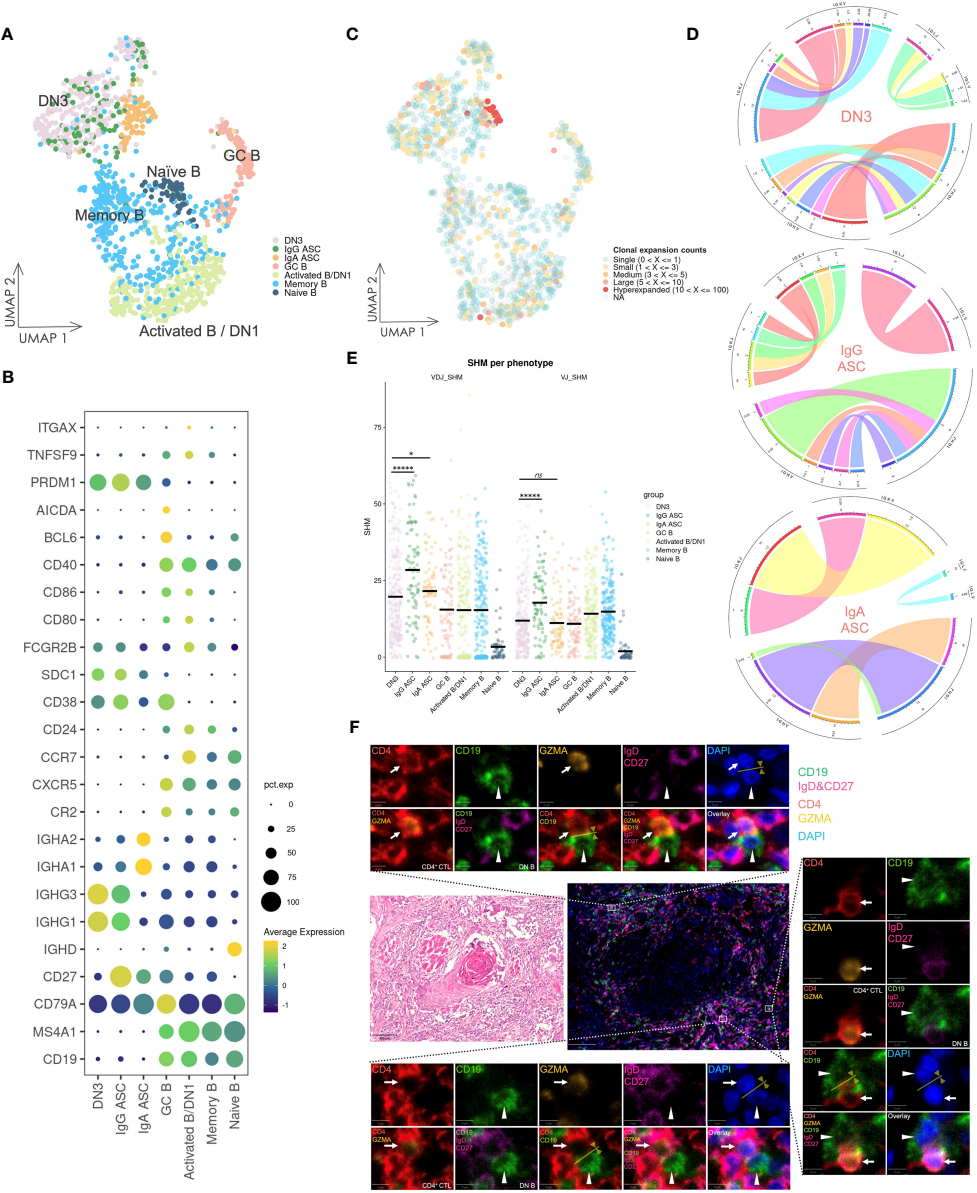
Gene expression and BCR repertoire analysis of B cells. **(A)** UMAP visualization of B cells. Cells on the UMAP are colored in accordance with the subsets. DN1: double negative 1 B cells; DN3: double negative 3 B cells; ASC: antibody-secreting cell; GC B: germinal center B cell. **(B)** Dot plot of gene expression in B cell subsets. **(C)** UMAP visualization of B cells integrated with BCR clonal expansion. The gradient color represents different degree of clonal expansion in distinct BCR clonotypes. NA: BCR sequence was undetected. **(D)** Circos plot of VJ germline gene usage in DN3 and ASC cells. This plot provided a circular visualization of how V and J germline genes were combined. **(E)** Visualization of the SHM frequency in B cell subsets. The black line on the dots represents the mean value. P-values were calculated by the Kruskal–Wallis test and adjusted by the Benjamini–Hochberg method, *P < 0.05, *****P < 0.00001, *ns*: not significant. **(F)** HE staining and multicolor IF staining of DN B cells and conjugation with CD4^+^ CTLs in two consecutive OSCC sections. Conjugation between these cells is depicted by the yellow arrow and line. Scale bars: 100 µm (low magnification) and 5 µm (high magnification).

Considering the role of CD4^+^ T cells in promoting B cell activation and differentiation, we examined the subsets of B cells and ASCs infiltrating into OSCC lesions (**Figures 5A, B**). Naïve B cells were identified by high *IGHD* expression and the absence of *CD27* expression, whereas memory B cells lacked *IGHD* expression. The activated B/DN1 subset exhibited *CXCR5* and low *CD27* expression but increased expression of *CCR7* and *CD40* compared with memory B cells. GC B cells were marked by expression of *BCL6* and *AICDA*. DN3 B cells lacked *IGHD*, *CD27*, *CXCR5, CR2*, and *ITGAX* encoding CD11c expression, whereas IgG and IgA ASCs exhibited high *IGHG* and *IGHA* expression, respectively. Although DN2 and DN4 subsets failed to form distinct clusters because of their scarcity in the scRNA-seq data, their presence was confirmed by multicolor IF staining (**Supplementary Figure 12**).

### 3.9 DN3 B cells synthesize IgG antibodies, potentially originating from extrafollicular responses, and frequently conjugate with CD4^+^ CTLs

DN3 B cells showed notably high expression of *IGHG1* and *IGHG3*, even compared with IgG ASCs (**Figure 5B**), and analysis of the BCR repertoire revealed oligoclonally expanded populations in activated B/DN1, DN3, and ASC subsets (**Figure 5C**, **Supplementary Figure 13**), whereas BCR clonal expansion in naïve B cells was almost single and BCR diversity and frequency of unique BCR clonotypes was high, indicating activation and differentiation of DN3 B cells. Subsequently, we selected B cells containing more than three counts per clonotype from the DN3 and ASC subsets and visualized VJ germline gene usage (**Figure 5D**). Higher variability was observed in the germline gene usage of DN3 B cells compared with that of ASC subsets, indicating diverse expanded clonotypes of DN3 B cells, which was a characteristic of extrafollicular responses.

Additionally, we investigated the frequency of SHMs across these B cell subsets in heavy chains and light chains (**Figure 5E**). DN3 B cells exhibited a significantly lower frequency of SHMs compared with IgG ASCs (adjusted p-values of 6.426e-06 and 5.355e-06 in heavy chains and light chains, respectively). This suggested that DN3 B cells were activated and differentiated primarily through extrafollicular responses.

To further examine the potential coactivation between DN B cells and CD4^+^ CTLs, we performed HE and multicolor IF staining of OSCC tissue samples (**Figure 5F**). Conjugation between DN B cells and CD4^+^ CTLs was observed in extrafollicular sites adjacent to tumor cells. Such conjugation implied that CD4^+^ CTLs facilitated DN B cell activation and promoted their differentiation via extrafollicular responses. Conversely, DN B cells may also further activate CD4^+^ CTLs in these responses.

### 3.10 Infiltration of CD4^+^ CTLs may imply a poor prognosis of OSCC

**Figure 6:**
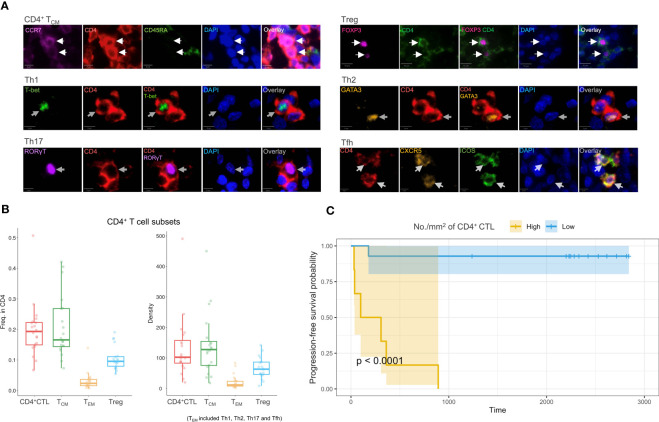
Multicolor IF staining and survival analysis. **(A)** Multicolor IF staining of CD4^+^ T cell subsets in OSCC tissue. The white arrow indicates cells in distinct subsets. Th1: T helper 1 cell; Th2: T helper 2 cell; Th17: T help 17 cell; Tfh: T follicular helper cell; Treg: regulatory T cell. Scale bars: 10 µm (low magnification) and 5 µm (high magnification). **(B)** Box plots of the frequency of CD4^+^ T cell subsets (*n* = 20) among CD4^+^ cells (left) and the density of these subsets (right). The T_EM_ subsets included Th1, Th2, Th17, and Tfh subsets. **(C)** Progression-free survival analysis using Kaplan–Meier models. The p-value was calculated by the log-rank test.

Multicolor IF staining and quantification of CD4^+^ T cell subsets were conducted in 20 tissue samples obtained from patients with OSCC of the tongue (**Figures 6A, B**). The quantification indicated substantial infiltration of CD4^+^ CTLs into OSCC lesions compared with other CD4^+^ T cell subsets. To elucidate the potential correlation between CD4^+^ T cell subsets and prognosis, patients were divided into two groups by the density of infiltrating CD4^+^ CTLs, and Kaplan–Meier models were constructed to compare the survival distribution (**Figure 6C**). The results showed statistical significance in progression-free survival probabilities between the two groups, indicating that patients with a large number of CD4^+^ CTLs may have a poor prognosis.

Considering potential covariates may affect prognosis, we constructed several COX proportional hazard models. Initially, we constructed singular COX models for each variable, which included the density of individual T cell subsets, age, sex, T and N classification, and differentiation of carcinoma to filter these variables. The density of CD4^+^ CTLs, Tregs, and Th2 and Tfh cells, as well as the N classification demonstrated statistical significance (**Supplementary Table 3**). We next constructed models that incorporated the density of each CD4^+^ T cell subset with the N classification (**Table 1**, **Supplementary Table 4**). A high density of CD4^+^ CTLs infiltrating into OSCC lesions was identified to be a significant risk factor potentially influencing progression-free survival time, whereas the density of Tregs, and Th2 and Tfh cells did not show significant results in these models.

**Table 1:** COX proportional hazard model of the CD4^+^CTL density and N classification.

	coef	HR	se(coef)	z	p-value	lower.95	upper.95
CD4^+^CTL	0.007807	1.008	0.003663	2.131	0.03308*	1.001	1.015
N1	3.396	29.84	1.399	2.427	0.01522*	1.922	463.096
N2a	3.582	35.94	1.647	2.175	0.02961*	1.426	905.934
N2b	4.424	83.44	1.784	2.480	0.01316*	2.527	2755.381
N3b	5.203	181.9	1.929	2.697	0.00698**	4.151	7971.556

coef, estimated coefficients; HR, hazard ratio; se(coef), standard error of the coefficient. *P < 0.05, **P < 0.01.

## 4 Discussion

CD4^+^ CTLs play a protective role against several chronic and acute viral infections, such as HIV, Epstein-Barr virus, and Dengue virus (40, 49, 50). We previously reported identification of this non-conventional CD4^+^ helper T cell subset as the dominant tissue-infiltrating CD4^+^ T cell subset driving inflammation in several autoimmune diseases including IgG4-related disease and systemic sclerosis (11, 51). Meanwhile, research by our group and others also indicated the expansion of CD4^+^ CTLs in the context of COVID-19 (10, 52). In addition to their significance in these diseases, the increased focus on CD4^+^ T cells and CD4^+^ CTLs in cancer research has come about because only a minority of tumors respond to current immunotherapies including immune checkpoint inhibitor therapy. Direct cytotoxicity of CD4^+^ CTLs against tumor cells was initially reported in melanoma patients treated with ipilimumab, a monoclonal antibody targeting CTLA-4. Subsequent investigations have gathered evidence supporting the indispensable role of CD4^+^ CTLs in tumor immunity for other cancers, such as lung, colorectal, and bladder cancers (53–56). Previous studies have mainly focused on the cytotoxicity of CD4^+^ CTLs to establish effective immunotherapy. In this study, we revealed that many CD4^+^ CTLs infiltrate the tumor lesion together with CD8^+^ T cells. Moreover, these CD4^+^ CTLs possess diverse functions other than their cytotoxic activity.

We first identified the CD4^+^ CTL subset as a unique subset of tumor-infiltrating immune cells in OSCC, characterized by a distinct gene expression pattern including the expression of cytotoxic genes through scRNA-seq analysis with strict quality control, TCR repertoire analysis and SCENIC analysis. Additionally, the results of single cell trajectories, TCR clonal expansion, and RNA velocity analysis increased our understanding of the dynamic processes underlying conventional CD4^+^ T cell development in OSCC tissue, while it would be limited in accuracy or confidence because of results based on calculations and predictions. We found that infiltrating CD4^+^ T_CM_ cells developed and acquired the capacity to produce CXCL13, which was facilitated by antigen-presenting cells. However, the state of these cells was changing quickly. Conditions of high antigen load and prolonged antigen exposure may contribute to differentiation into CD4^+^ CTLs, a cell state that was more stable but represented a terminal stage of differentiation. Subsequently, we examined the potential cytotoxicity of CD4^+^ CTLs. We found that tumor cells expressing MHC class II molecules may be directly eliminated by CD4^+^ CTLs, potentially via degranulation and perforin release or activation of the TRAIL–TRAIL receptor axis.

Application of high dimensional WGCNA to the CD4^+^ CTL subset revealed similarities in the expression of hMEs in modules between CD4^+^ CTLs and Tregs, suggesting that CD4^+^ CTLs likely possess immune regulatory functions despite their differing lineage. Considering that these CD4^+^ CTLs are the most terminally developed subset among all conventional CD4^+^ T cell subsets, coupled with their cytotoxic activity and potential to suppress B cell responses via CTLA-4 expression, CD4^+^ CTLs may mediate immunosuppression and tolerance functions in both homeostasis and inflammation.

An interesting finding was a module containing IR genes upregulated in CD4^+^ CTL, Treg, and CD8^+^ T_EX_ subsets. Paired with upregulated expression of IR genes, such as *PDCD1, LAG3, TIGIT, HAVCR2*, and *CTLA4*, this result suggests that cells in the CD4^+^CTL 2 subset were exhausted or dysfunctional in OSCC, although contention remains on whether markers of CD8^+^ T cell exhaustion can be applied to their CD4^+^ counterparts (57). This speculation was further corroborated by high *CXCL13*, *ENTPD1*, and *TOX* expression, and lost *TCF7* expression in the CD4^+^CTL 2 subset (48, 58). Considering that CD4^+^ CTLs are characterized as a terminally developed phenotype of CD4^+^ T cells and the presence of CD4^+^ T cell exhaustion phenotype is reported in several solid tumors, it is possible that these cells are exhausted or dysfunctional CD4^+^ T cells and contribute to tumor progression via immunosuppression (59, 60). In fact, infiltration of CD4^+^ CTLs appeared to lead to poor outcomes of OSCC patients, as observed in our retrospective evaluation of clinicopathological parameters. Chen et al. demonstrated that CTLA-4 blockade can promote the tumor immunity through a CD4^+^ T cell-dependent manner in glioblastoma (61). Likewise, Mccaw et al. showed that CD4^+^ CTLs initially delay tumor growth, but progression ultimately becomes uncontrolled, together with expression of coinhibitory molecules in murine breast tumor. Crucially, this phenomenon was reversed through anti-CTLA4 therapy (60). Both studies underscore the role of CD4^+^ T cell exhaustion in tumor progression and highlight the potential of CD4-targeted therapy.

Another interesting finding was that CD4^+^ CTLs expressed B cell-attracting chemokine CXCL13, thereby inducing B cell migration to tumor lesions. In general, CXCL13 is typically expressed by HEVs and follicular stromal cells in the B cell region of secondary lymphoid organs. Nevertheless, several studies have reported CXCL13-producing CD4^+^ T cells, which have been associated with favorable outcomes of tumor immunity (62–64). Despite such findings, the effects of CXCL13 expression on clinical outcomes remain controversial, particularly in terms of the association between high infiltration of CXCL13^+^ CD8^+^ T cells in tumor lesions and poor clinical outcomes (65, 66). Regardless, the finding that CD4^+^ CTLs are predisposed to invoke B cells and mediate humoral immunity through CXCL13 expression emphasizes their central role in tumor immunity in addition to their CD8^+^ CTL counterparts.

Following this finding, we revealed expansion of activated B cells, including DN3 B cells. DN3 B cells have been identified as extrafollicular B cells in several immune conditions including IgG4-related disease and COVID-19; these conditions are accompanied by CD4^+^ CTL enlargement (67, 68). In OSCC, expanded CD4^+^ CTLs physically interacted with these DN B cells in extrafollicular regions, a scenario we have previously reported in other diseases. We observed clonal expansion and markedly higher expression of IgG-related genes in DN3 B cells. Additionally, the diverse VJ germline gene usage and lower frequency of SHMs suggested that DN3 B cells may arise from extrafollicular responses and lack affinity maturation. Considering that the affinity of IgG antibodies is likely to be low, whether the antibodies produced by DN3 B cells are associated or specific to tumor remains to be determined, which warrants further investigation.

These seemingly contradictory effects suggested heterogeneity in the CD4^+^ CTL subset. This heterogeneity may be due to the different exhausted states and origins of distinct T cell subsets (69). The question arises as to why such a specific immune microenvironment is observed in tumor lesions. A potential explanation is related with the resemblance of the tumor environment to T and B cell-activated diseases. In such environment, immune cells are constantly exposed to damage-associated molecular patterns and neoantigens, causing sustained activation of T and B cells, especially in ‘hot’ tumors. Our previous research across several disease situations, often characterized by high cytokine production and lymphocyte activation, suggests that such circumstances foster an extrafollicular response, subsequently instigating differentiation of T and B cells into CD4^+^ CTLs and DN3 B cells, respectively. A notable example is COVID-19, in which cytokine storms represent such a typical disease (9, 68). Research deciphering these specific immune responses may ultimately deepen our comprehension concerning the underlying immune mechanisms of a broad spectrum of T and B cell-activated diseases including autoimmune and infectious diseases. However, there are several limitations to this study. The data presented here is descriptive and suggestive of the tumor immunity mechanism focused on CD4^+^ CTLs. Since we examined samples from human patients rather than a mouse lab model, we could not explore the mechanism through which CD4^+^ T cell differentiation and CD4^+^ CTLs involvement in tumor immunity occurred in the patients. Furthermore, although we found several clonal expanded TCRs, we cannot identify those targets; this is one of the factors that made the mechanism challenging to ascertain in this study. Further studies should address these points.

**Figure 7:**
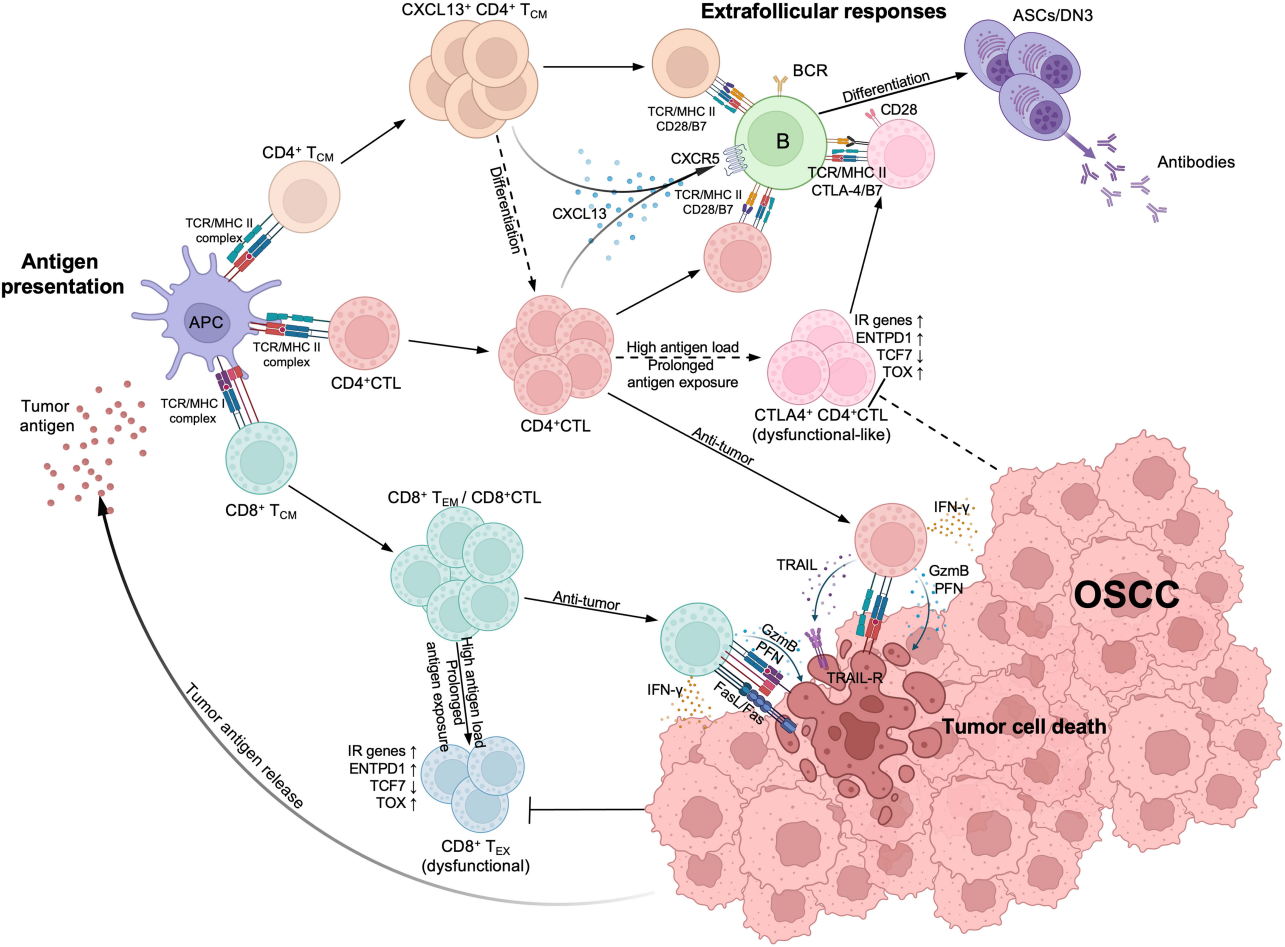
Estimated schema of CD4^+^CTL and other T cells immunological processes in oral squamous cell carcinoma (OSCC). (Top left) Upon tumor antigen presented by antigen-presenting cells, activated CD4^+^ cytotoxic T lymphocytes (CD4^+^CTL), CD4^+^ T_CM_ and CD8^+^ T_CM_ expanded within tumor lesions. CD8^+^ T_CM_ subsequently differentiates into effector memory T cell (CD8^+^ T_EM_) or CD8^+^CTL and expresses strong cytotoxicity. (Bottom right) Both CD4^+^CTLs and CD8^+^CTLs can induce tumor cell death; CD4^+^CTLs recognize the antigens presented via MHC class II molecules (MHC II) and induce the tumor cell death through granzyme B (GzmB) and perforin (PFN) release or TRAIL release, while CD8^+^CTLs also express Fas ligand (FasL) and recognize antigens presented via MHC class I molecules (MHC II). (Top right) Activated CD4^+^ T_CM_ and CD4^+^CTLs produce CXCL13, attracting CXCR5^+^ B cells and interacting with them via TCR/MHC class II and costimulators such as CD28/B7, thereby initiating extrafollicular responses and promoting the differentiation of B cells into antibody secreting cells (ASCs) or double negative B cell 3 (DN3). IFN-γ produced by CD4^+^CTLs can also promote the responses. With high antigen load and prolonged antigen exposure, CD8^+^CTLs differentiate into CD8^+^ exhausted T cells (CD8^+^ T_EX_) and become dysfunctional, marked by upregulation of inhibitory receptor (IR) genes, *ENTPD1* and *TOX*, and the downregulation of *TCF7*. Although CD4^+^CTLs display similar gene expression patterns, whether they can become dysfunctional remains controversial (broken arrows). These CD4^+^CTLs also express CTLA-4, competitively binding with B7 molecules and potentially limiting immune responses between CD4^+^ T cells and B cells.

In conclusion, our study indicates that expanded CD4^+^ CTLs eliminate tumor cells by exerting their cytotoxicity, while possibly attenuating T and B cell activity or acquiring an exhausted or dysfunctional phenotype by expressing IRs and inhibitory molecules. This suggests a dual role of CD4^+^ CTLs in either promoting or suppressing tumor immunity, although the latter function is more prominent in OSCC. Furthermore, we observed that CD4^+^ CTLs produce CXCL13 that potentially interacts with activated B cell populations, including DN B cells, through physical conjugation in tumor lesions. We employed a schema to summarize these results (**Figure 7**). Whether CD4^+^ CTLs collaborate with DN3 B cells in tumor immunity remains undetermined, despite these observations having been consistently identified in the pathogenesis of IgG4-RD, systemic sclerosis, fibrosing mediastinitis, and COVID-19. Considering the effect of CD4^+^ CTLs in suppressing other cancers, including melanoma, breast cancer, bladder cancer, and colorectal cancer via their cytotoxicity, whether the dual role of CD4^+^ CTLs we found in this study is specific to OSCC needs to be verified in the future. Our results indicate the need for further investigation into the role of CD4^+^ CTLs and DN B cells as potential drivers or inhibitors of tumor immunity, which could enable the development of a novel cancer immunotherapeutic strategy.

## Data availability statement

Sequence data presented in the study are deposited in National Center for Biotechnology Information GEO database (https://www.ncbi.nlm.nih.gov/geo/), accession number GSE247582 and in BioProject, accession number PRJNA1039158.

## Ethics statement

The study design and methods were approved by the Institutional Review Board of the Center for Clinical and Translational Research of Kyushu University Hospital (IRB number: 834-00). The studies were conducted in accordance with the local legislation and institutional requirements. The participants provided their written informed consent to participate in this study.

## Author contributions

HC: Data curation, Formal Analysis, Investigation, Methodology, Validation, Writing – original draft, Writing – review & editing. JS: Resources, Writing – review & editing, Formal Analysis, Investigation, Methodology. SY: Investigation, Resources, Writing – review & editing. TSu: Investigation, Resources, Writing – review & editing. HN: Writing – review & editing, Formal Analysis. YM: Writing – review & editing, Resources. TSa: Resources, Writing – review & editing. SF: Resources, Writing – review & editing. TK: Resources, Writing – review & editing. TG: Data curation, Writing – review & editing. SN: Resources, Supervision, Writing – review & editing. MM: Conceptualization, Resources, Supervision, Writing – review & editing. NK: Conceptualization, Funding acquisition, Project administration, Resources, Supervision, Writing – original draft, Writing – review & editing. SK: Conceptualization, Resources, Supervision, Writing – review & editing.
