# Supplementary material for: Expansion of CD4+ cytotoxic T lymphocytes with specific gene expression patterns may contribute to suppression of tumor immunity in oral squamous cell carcinoma: single-cell analysis and in vitro experiments
Source: Front Immunol. 2023 Nov 23;14:1305783. doi: 10.3389/fimmu.2023.1305783 (PMC10702345; doi:10.3389/fimmu.2023.1305783)
Supplement: Supplementary file 1 [file DataSheet_1.pdf]

Supplementary Figure 1

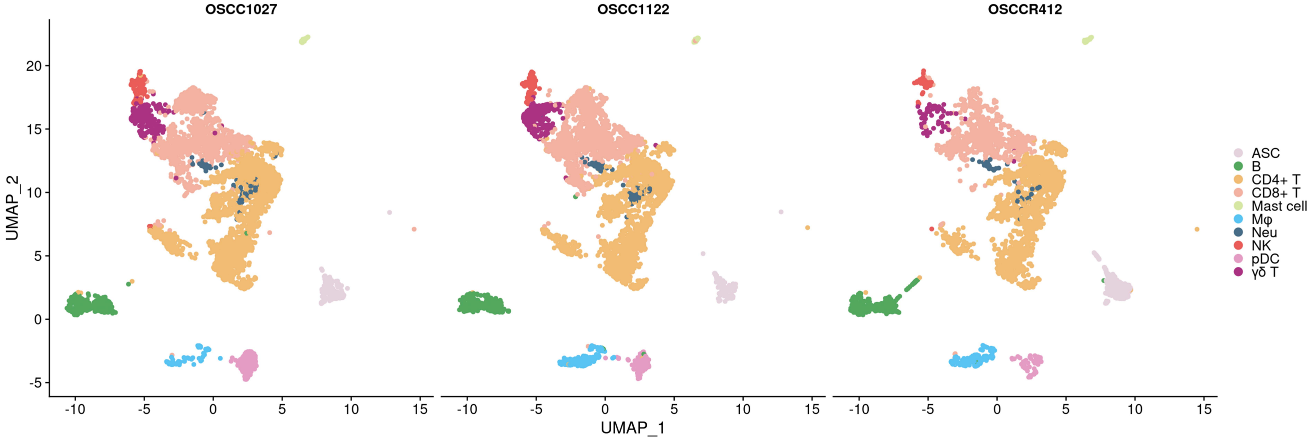

Supplementary Figure 1. UMAP visualization of the infiltrating immune cells split by the origin of tissue. ASC: antibody secreting cell; B: B cell; $CD4^+$ T: $CD4^+$ T cell; $CD8^+$: $CD8^+$ T cell; γδ T: γδ T cell; Mφ: macrophage; Neu: neutrophil; NK: natural killer cell; pDC: plasmacyotid dendritic cell.

Supplementary Figure 2

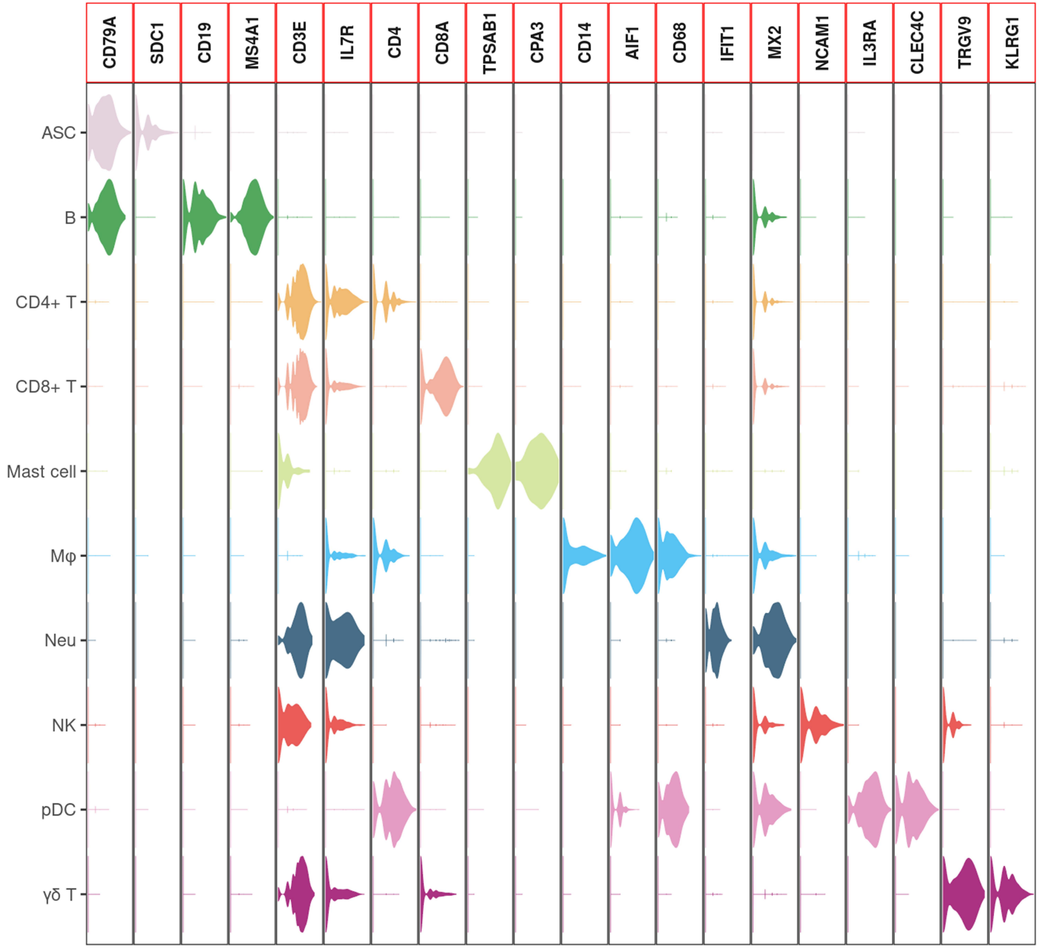

Supplementary Figure 2. Violin plot of the marker gene expression in immune cell subsets. The dataset with multiple SCT models were normalized to a comparable sequencing depth.

Supplementary Figure 3

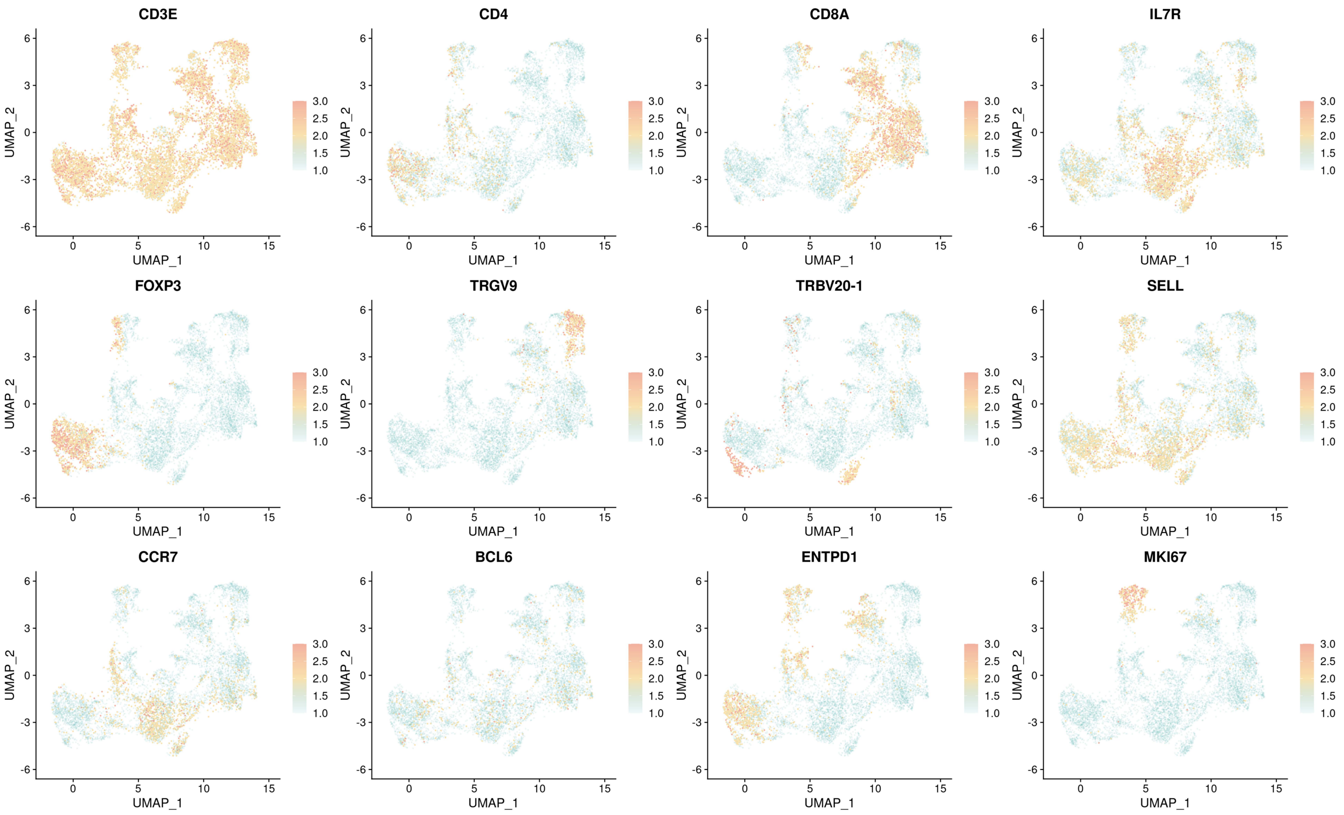

Supplementary Figure 3. Feature plot of marker gene expression in T cells. The dataset with multiple SCT models were normalized to a comparable sequencing depth.

Supplementary Figure 4

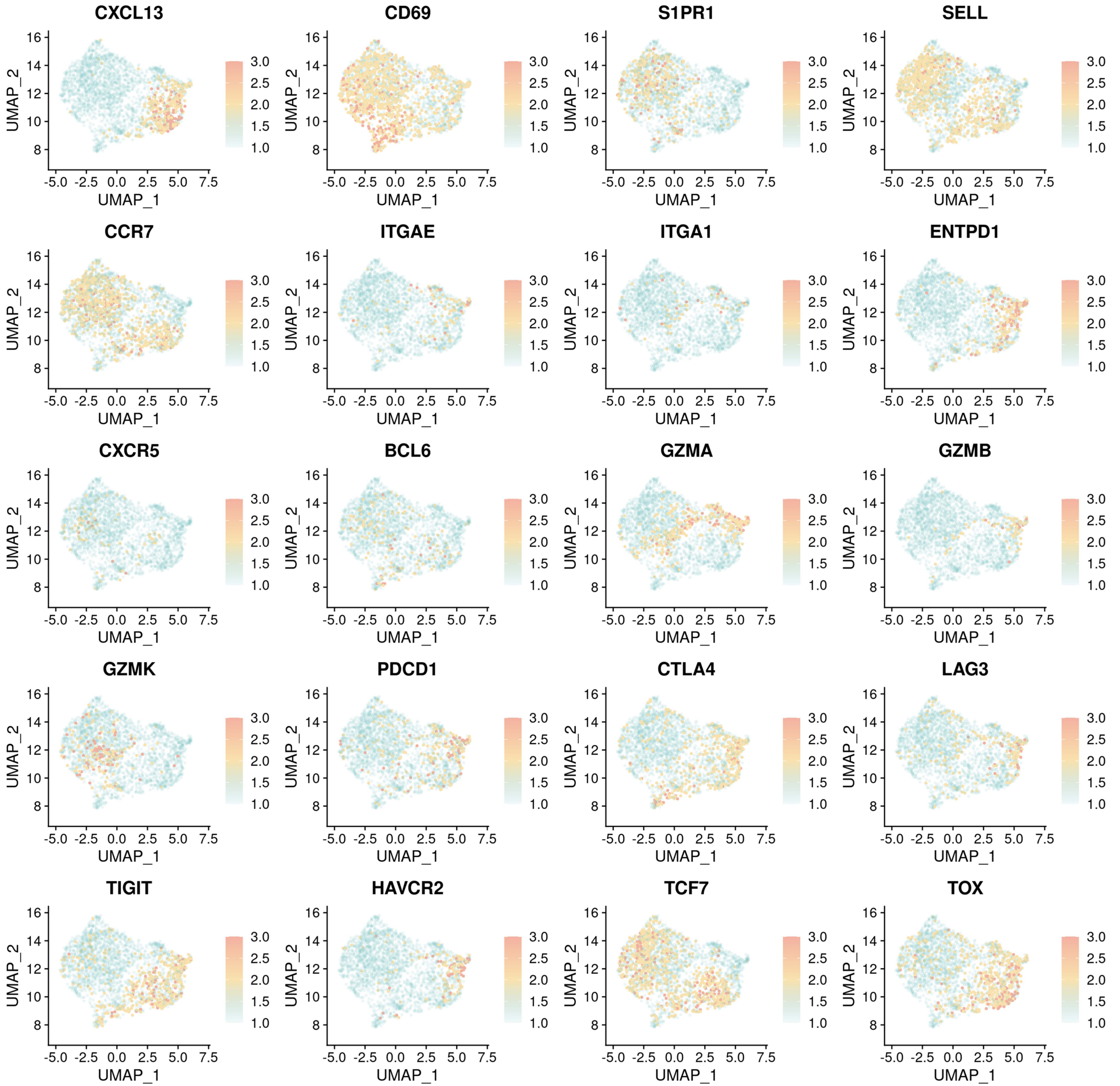

Supplementary Figure 4. Feature plot of gene expression in CD4^{+} T cells. The dataset with multiple SCT models were normalized to a comparable sequencing depth.

Supplementary Figure 5

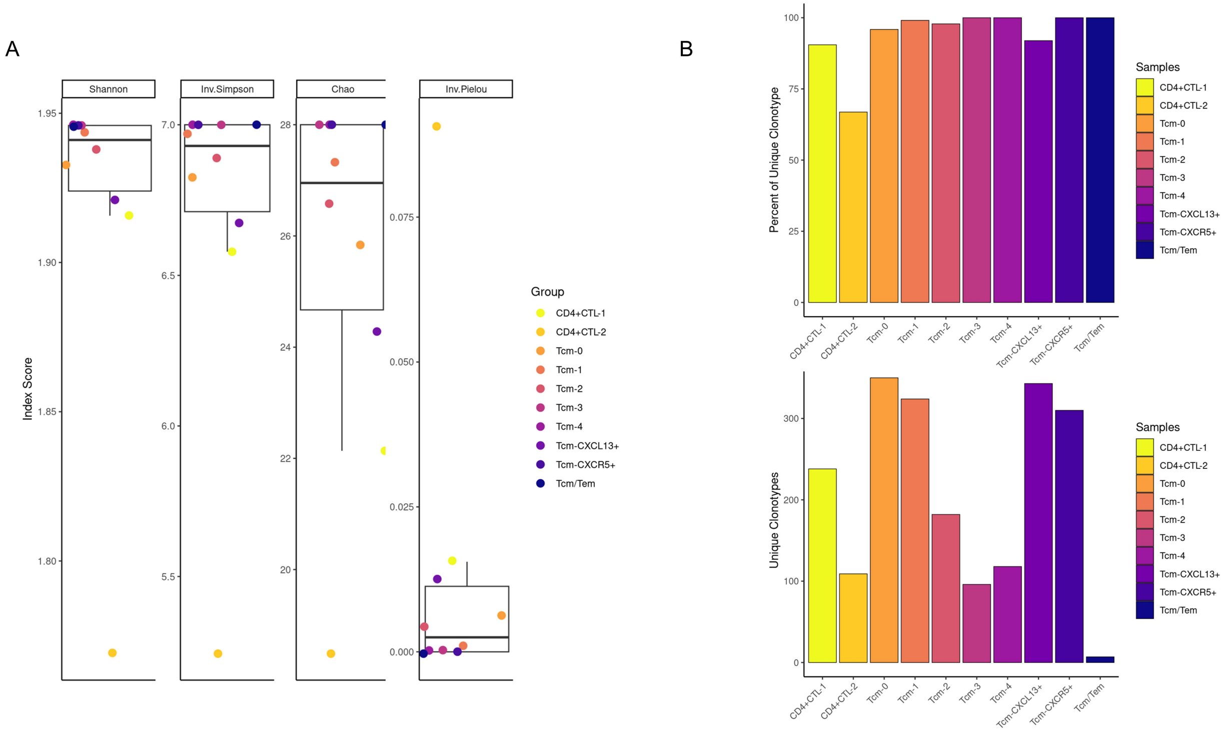

Supplementary Figure 5. Diversity estimation and unique clonotypes of conventional CD4$^+$ T cells. **(A)** Scores of Shannon Index, inverse Simpson Index, Chao1 Index and Pielou Index in conventional CD4$^+$ T cells. These indices provide an estimation of diversity in distinct conventional CD4$^+$ T cell subsets. **(B)** Percent of unique clonotypes and total number of unique clonotypes in these conventional CD4$^+$ T cell subsets

Supplementary Figure 6

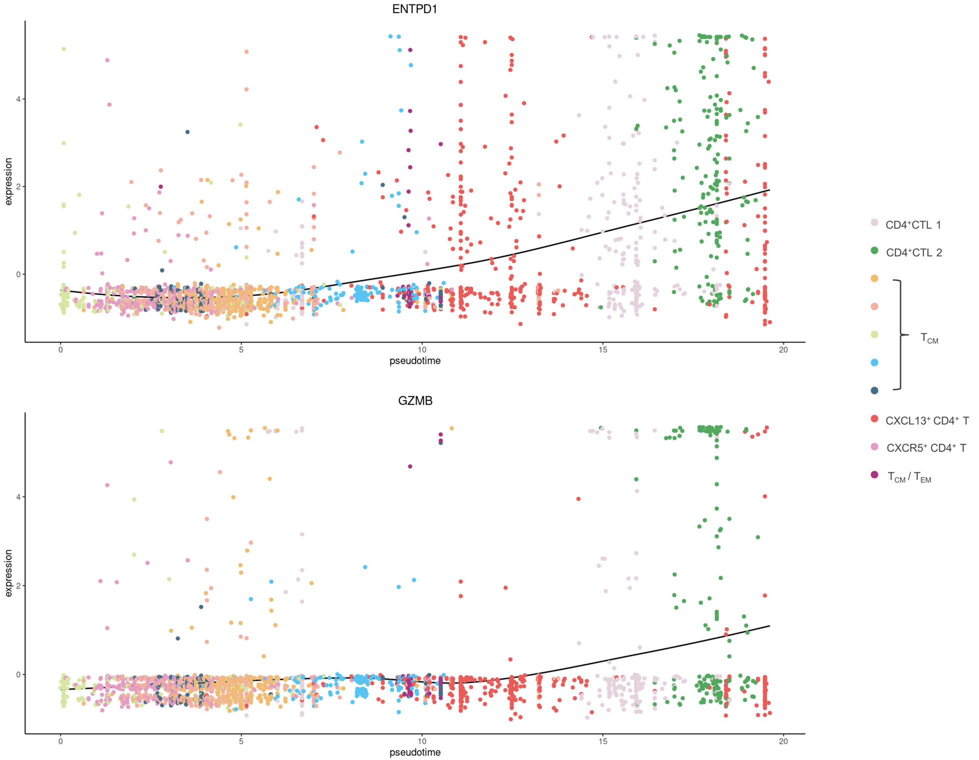

Supplementary Figure 6. Gene dynamic plot based on the pseudotime in conventional $CD4^+$ T cell subsets. The cells were ordered according to the pseudotime, and the average gene expression of these genes in cells with identical pseudotime was calculated and visualized in this plot. The dots were colored by different conventional $CD4^+$ T cell clusters.

Supplementary Figure 7

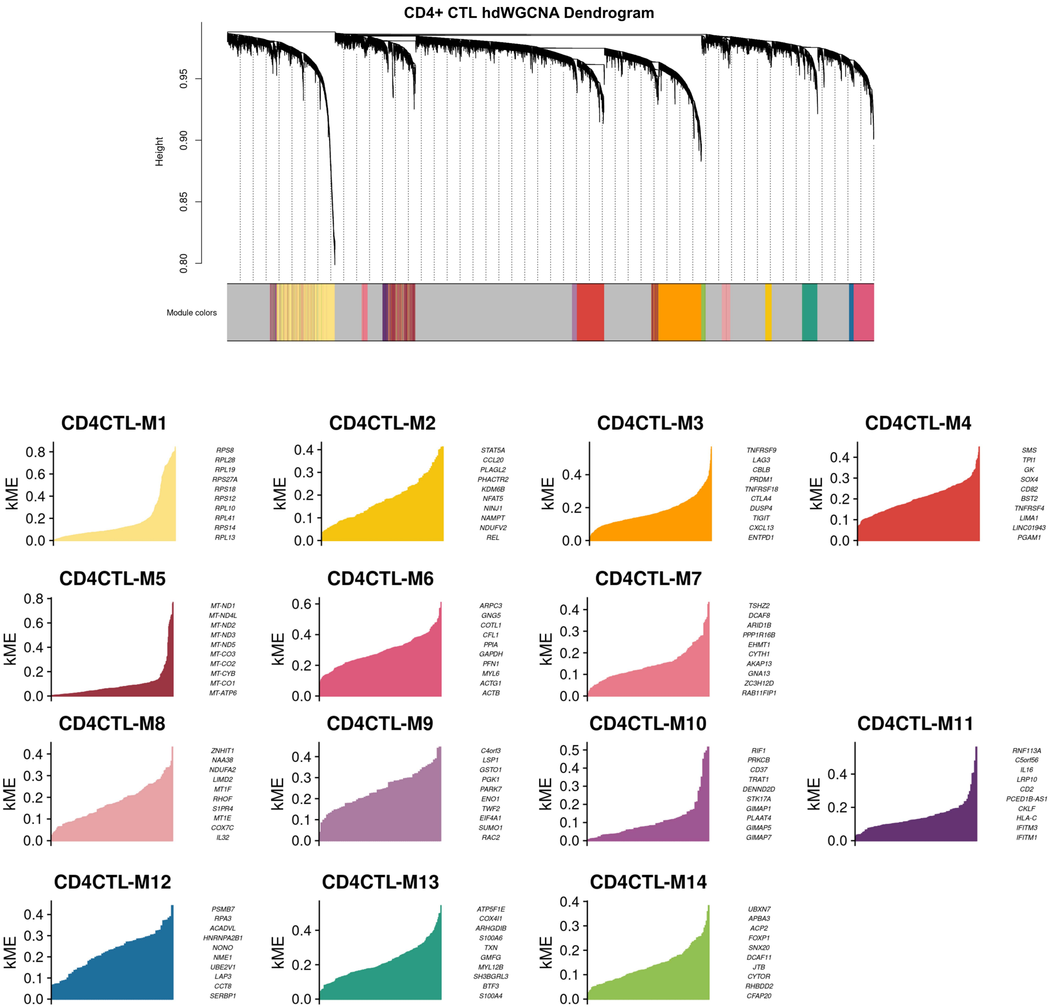

Supplementary Figure 7. High dimensional dendrogram for $CD4^{+}$CTL subset and visualization of genes in modules. The kME value for the determination of eigengene-based connectivity was used to order the genes in these modules and the top 10 kME value genes were displayed in the plots.

Supplementary Figure 8

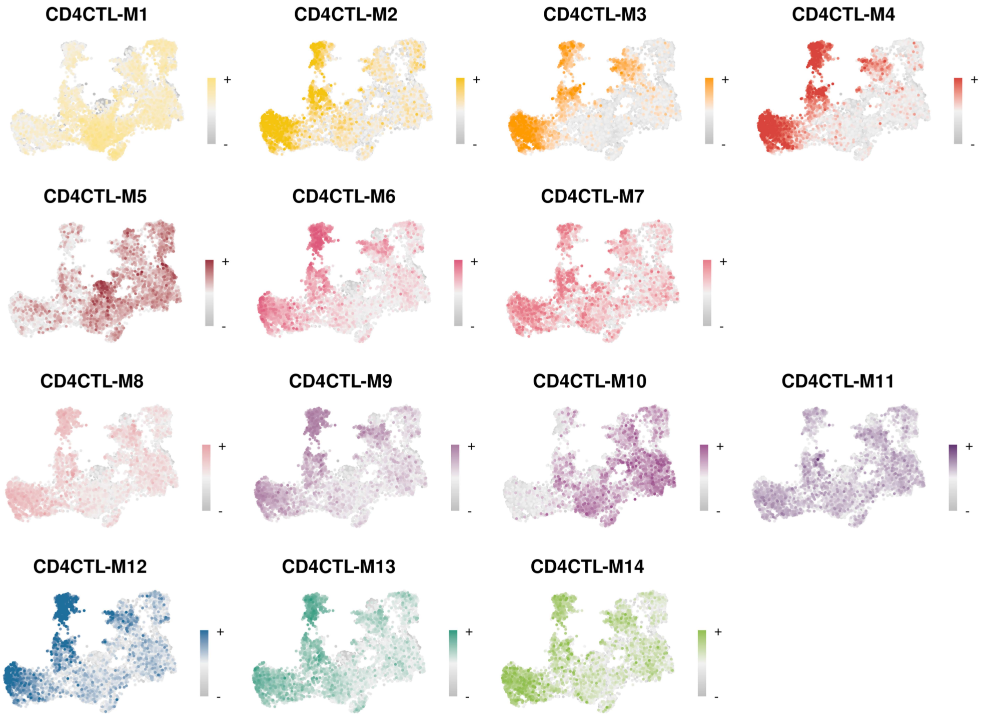

Supplementary Figure 8. Feature plot of hMEs in the modules. The expression of hMEs in these modules was computed in single cells and visualized on UMAP.

Supplementary Figure 9

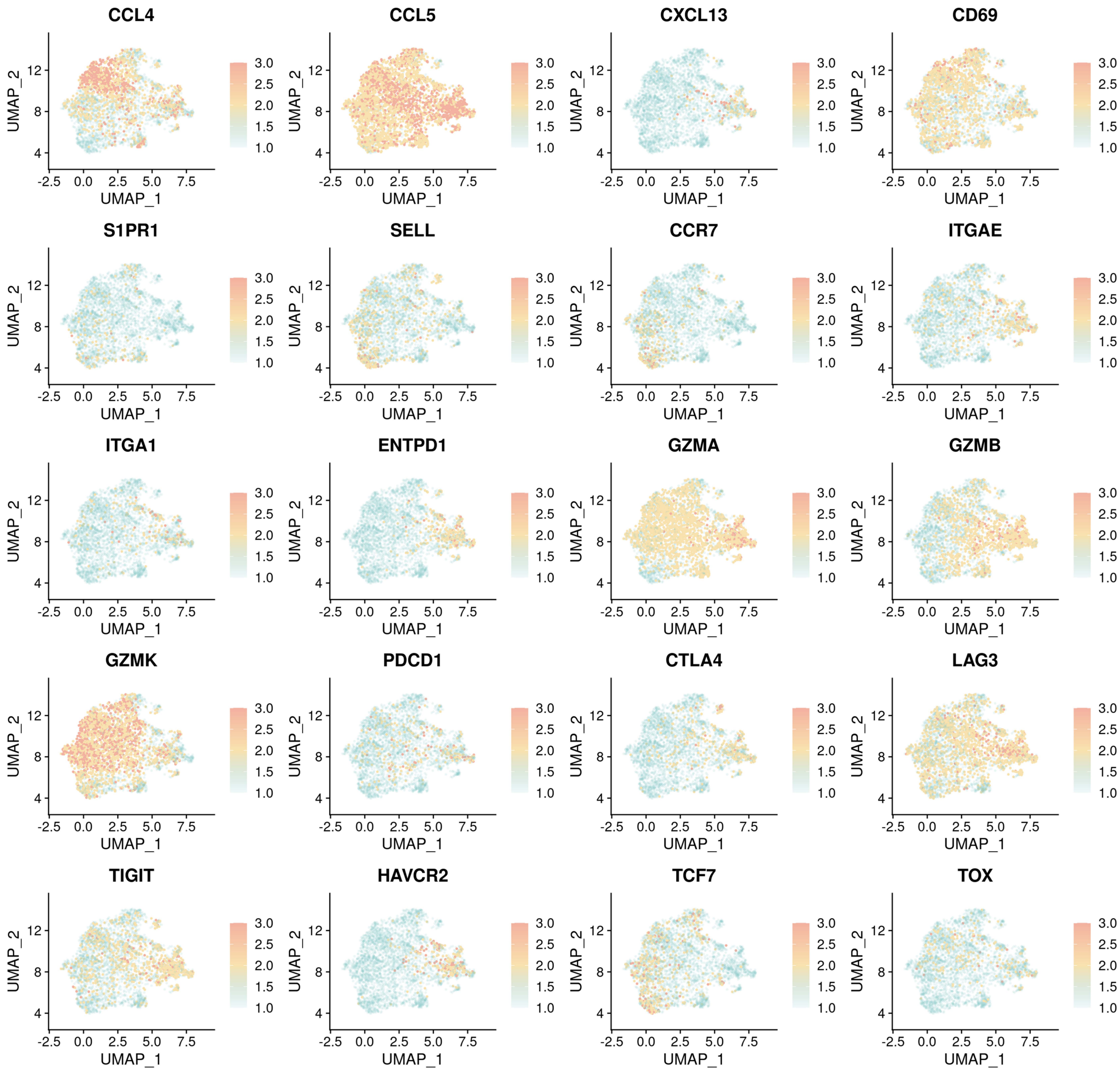

Supplementary Figure 9. Feature plot of gene expression in $CD8^+$ T cells. The dataset with multiple SCT models were normalized to a comparable sequencing depth.

Supplementary Figure 10

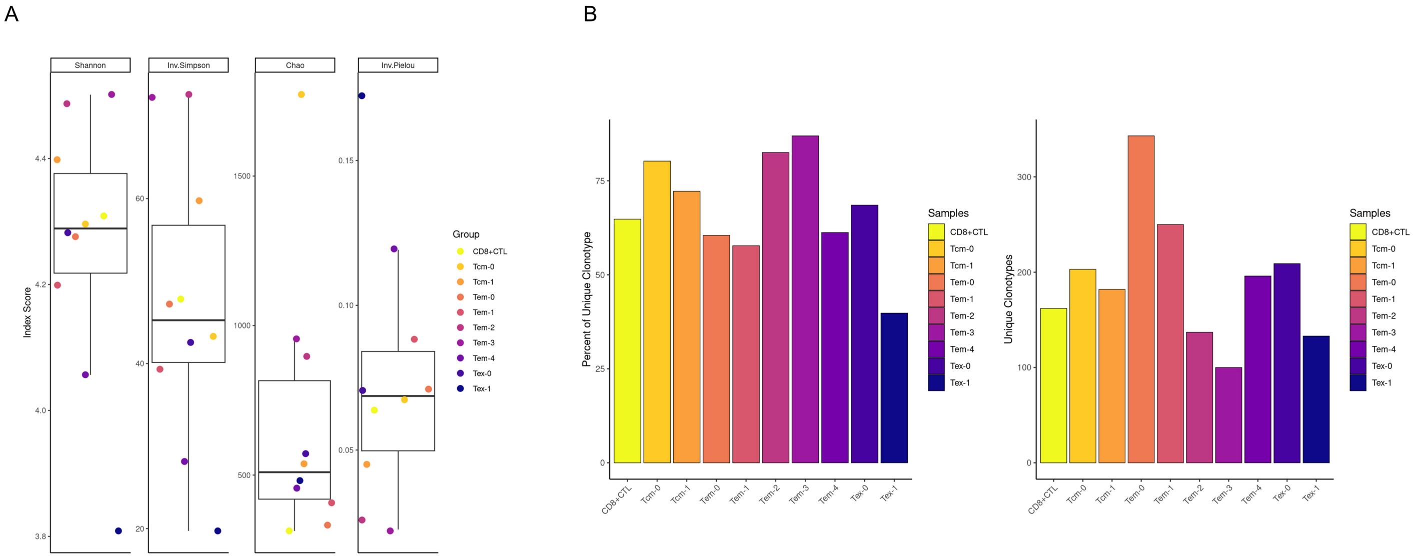

Supplementary Figure 10. Diversity estimation and unique clonotypes of CD8^{+} T cells. **(A)** Scores of Shannon Index, inverse Simpson Index, Chao1 Index and Pielou Index in CD8^{+} T cells. These indices provide an estimation of diversity in distinct CD8^{+} T cell subsets. **(B)** Percent of unique clonotypes and total number of unique clonotypes in these CD8^{+} T cell subsets

Supplementary Figure 11

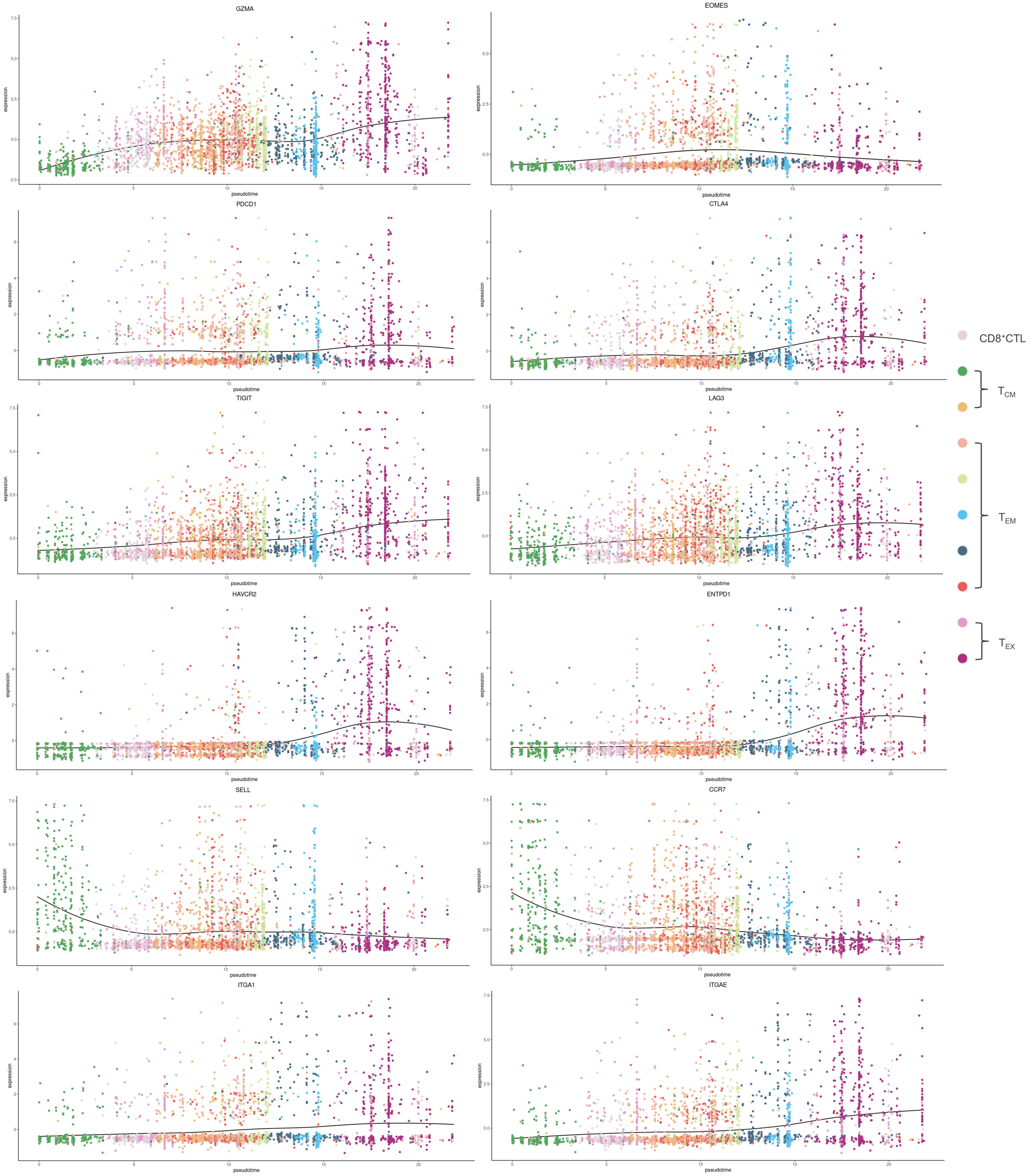

Supplementary Figure 11. Gene dynamic plot based on the pseudotime in CD8$^+$ T cell subsets. The cells were ordered according to the pseudotime, and the average gene expression of these genes in cells with identical pseudotime was calculated and visualized in this plot. The dots were colored by different CD8$^+$ T cell clusters.

Supplementary Figure 12

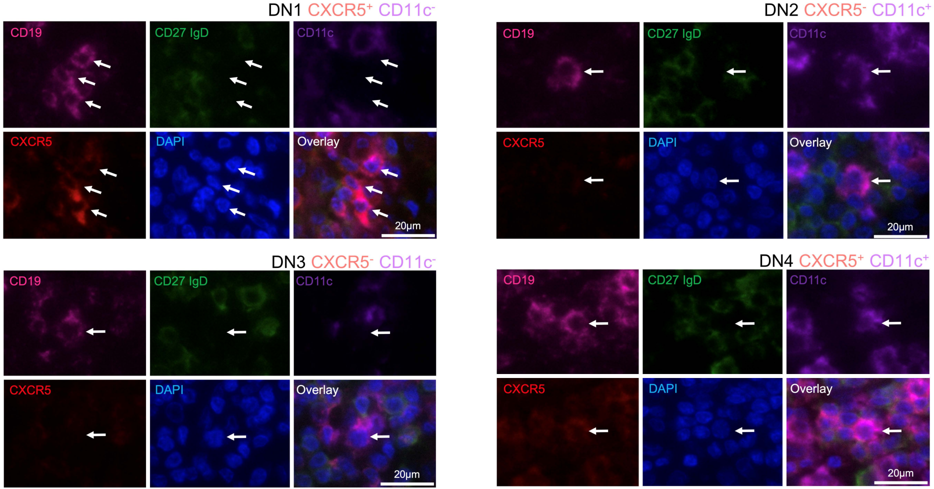

Supplementary Figure 12. Multi-color IF staining of DN B cell subsets in OSCC tissue. The white arrow showed the cells in distinct subsets. Scale bars: 20 μm.

Supplementary Figure 13

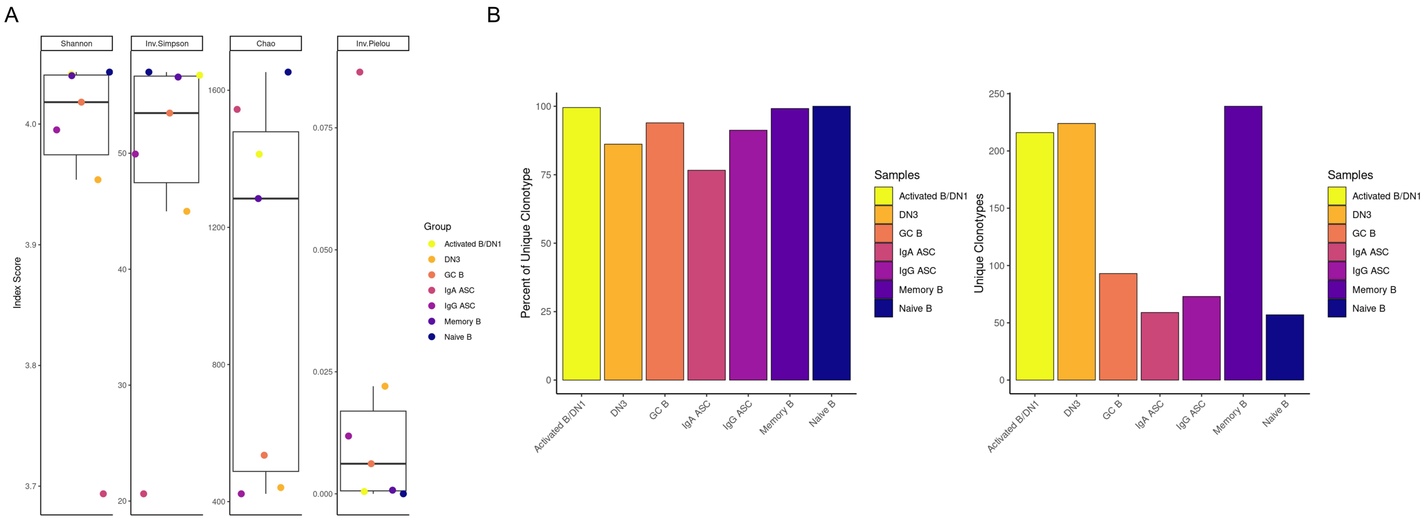

Supplementary Figure 13. Diversity estimation and unique clonotypes of B cells. **(A)** Scores of Shannon Index, inverse Simpson Index, Chao1 Index and Pielou Index in B cells. These indices provide an estimation of diversity in distinct B cell subsets. **(B)** Percent of unique clonotypes and total number of unique clonotypes in these B cell subsets

Supplementary Table 1. Patient information

Patient	Sex	Age	Differentiation of OSCC	TNM classification	Stage	Follow-up period (month)	Progression-free period (month)	Clinical outcomes	Application
P1	Male	32	well	T3N0M0	III	88	88	survival	multi-color IF, K-M and COX analysis
P2	Female	61	moderately	T2N0M0	II	93	75	survival	multi-color IF, K-M and COX analysis
P3	Female	60	well	T1N2aM0	IV	84	11	survival	multi-color IF, K-M and COX analysis
P4	Male	56	well	T2N2bM0	IV	9	4	death (distant metastasis)	multi-color IF, K-M and COX analysis
P5	Female	73	well	T1N0M0	I	78	30	survival	multi-color IF, K-M and COX analysis
P6	Male	45	well	T1N0M0	I	77	77	survival	multi-color IF, K-M and COX analysis
P7	Female	54	poorly	T3N3bM0	IV	60	2	survival	multi-color IF, K-M and COX analysis
P8	Male	56	well	T2N0M0	II	79	79	survival	multi-color IF, K-M and COX analysis
P9	Female	51	well	T3N0M0	III	42	42	survival	multi-color IF, K-M and COX analysis
P10	Male	85	well	T1N0M0	I	85	85	survival	multi-color IF, K-M and COX analysis
P11	Male	60	well	T1N0M0	I	92	91	survival	multi-color IF, K-M and COX analysis
P12	Female	69	well	T3N0M0	III	95	94	survival	multi-color IF, K-M and COX analysis
P13	Male	34	well	T2N0M0	II	75	75	survival	multi-color IF, K-M and COX analysis
P14	Male	66	moderately	T2N0M0	II	96	96	survival	multi-color IF, K-M and COX analysis
P15	Female	50	Early invasive carcinoma	T2N0M0	II	75	75	survival	multi-color IF, K-M and COX analysis
P16	Male	70	well	T2N1M0	III	83	12	survival	multi-color IF, K-M and COX analysis
P17	Male	64	Carcinoma in situ	TisN0M0	0	82	81	survival	multi-color IF, K-M and COX analysis
P18	Male	80	moderately	T2N0M0	II	74	74	survival	multi-color IF, K-M and COX analysis
P19	Male	73	moderately	T2N1M0	III	85	6	survival	multi-color IF, K-M and COX analysis
P20	Female	60	moderately	T3N0M0	III	39	2	death (unrelated causes)	multi-color IF, K-M and COX analysis
P21	Male	23	well	T3N0M0	III	6	6	survival	scRNA-seq
P22	Male	80	well	T2N0M0	II	13	4	survival	scRNA-seq
P23	Male	79	well	T4aN1M0	IV	12	9	survival	scRNA-seq

Supplementary Table 2. Antibody information

Antibodies	Description	Source	Identifier	Concen-tration	Antigen Retrieval	Incubating Conditions
Anti-CD45	Rabbit monoclonal	Cell Signaling	#13917	1:100	pH 6	Overnight at 4 °C
Anti-CD4	Rabbit monoclonal	Abcam	ab133616	1:500	pH 9	Overnight at 4 °C
Anti-Granzyme A	Rabbit monoclonal	Abcam	ab209205	1:100	pH 9	2 hours at RT
Anti-BCA1	Rabbit monoclonal	Abcam	ab246518	1:400	pH 9	2 hours at RT
Anti-CTLA4	Rabbit monoclonal	Abcam	ab237712	1:400	pH 9	2 hours at RT
Anti-CD19	Mouse monoclonal	Biocare Medical	310A	1:100	pH 6	Overnight at 4 °C
Anti-CD20	Rabbit monoclonal	Abcam	ab78237	1:300	pH 9	Overnight at 4 °C
Anti-CD27	Rabbit monoclonal	Abcam	ab131254	1:500	pH 9	1 hour at RT
Anti-IgD	Rabbit polyclonal	Dako	A0093	1:2000	pH 9	1 hour at RT
Anti-CD11c	Rabbit monoclonal	Abcam	ab52632	1:300	pH 9	Overnight at 4 °C
Anti-CXCR5	Rabbit monoclonal	Abcam	ab254415	1:2000	pH 6	2 hours at RT
Anti-CD39	Rabbit monoclonal	Abcam	ab223842	1:1000	pH 9	Overnight at 4 °C
Anti-CD8α	Rabbit monoclonal	Cell Signaling	#85336	1:200	pH 9	2 hours at RT
Anti-PDCD1	Mouse monoclonal	Lifespan Biosciences	LS-B13300	1:100	pH 6	1 hour at RT
Anti-pan cytokeratin	Mouse monoclonal	Abcam	ab27988	1:200	pH 6	1 hour at RT
Anti-HLA-DR	Mouse monoclonal	Abcam	ab20181	1:500	pH 6	Overnight at 4 °C
Anti-CCR7	Rabbit monoclonal	Abcam	ab253187	1:100	pH 6	Overnight at 4 °C
Anti-CD45RA	Mouse monoclonal	Abcam	ab755	1:500	pH 9	1 hour at RT
Anti-T-bet/Tbx21	Rabbit monoclonal	Cell Signaling	#13232	1:200	pH 6	Overnight at 4 °C
Anti-GATA3	Rabbit monoclonal	Abcam	ab199428	1:200	pH 9	2 hours at RT
Anti-RORγT	Mouse monoclonal	Biocare Medical	3208A	1:100	pH 6	Overnight at 4 °C
Anti-ICOS	Rabbit monoclonal	Abcam	#89601	1:200	pH 6	2 hours at RT
Anti-FoxP3	Rabbit monoclonal	Cell Signaling	#98377	1:100	pH 6	2 hours at RT
Anti-Granzyme B	Rabbit polyclonal	Abcam	ab4059	1:100	pH 6	Overnight at 4 °C
Anti-Cleaved Caspase-3	Rabbit polyclonal	Cell Signaling	#9661	1:100	pH 6	Overnight at 4 °C

Supplementary Table 3. Summary of COX proportional hazard models for each variable

	coef	HR	se(coef)	z	p-value	lower .95	upper .95
$CD4^{+}CTL$	0.005	1.005	0.002	2.303	0.021	1.001	1.009
T_{CM}	-0.002	0.998	0.004	-0.492	0.623	0.990	1.006
Th1	0.082	1.085	0.054	1.505	0.132	0.976	1.207
Th2	0.043	1.044	0.020	2.100	0.036	1.003	1.086
Th17	-0.032	0.968	0.148	-0.217	0.828	0.724	1.295
Tfh	0.135	1.145	0.067	2.008	0.045	1.003	1.306
Treg	0.021	1.022	0.011	2.004	0.045	1.000	1.043
Age	0.020	1.020	0.028	0.710	0.478	0.966	1.078
Sex: Male	-0.865	0.421	0.765	-1.131	0.258	0.094	1.885
T: Tis	-18.08	1.403e-08	12900	-0.001	0.999	0.000	Inf
T: T2	-0.098	0.907	0.914	-0.107	0.915	0.151	5.435
T: T3	0.330	1.391	1.002	0.329	0.742	0.195	9.916
N: N1	3.032	20.73	1.237	2.451	0.014	1.835	234.2
N: N2a	3.160	23.58	1.500	2.107	0.035	1.247	445.7
N: N2b	4.373	79.29	1.717	2.547	0.011	2.742	2293
N: N3b	5.068	158.8	1.855	2.732	0.006	4.186	6027
D: Moderately	0.591	1.807	0.867	0.682	0.495	0.330	9.889
D: Poorly	3.906	22.11	1.450	2.136	0.033	1.29	379.0

Note: coef: estimated coefficients; HR: hazard ratio; se(coef): standard error of the coefficient; D: differentiation of carcinoma

Supplementary Table 4. Summary of COX proportional hazard models for different combination of variables

	coef	HR	se(coef)	z	p-value	lower .95	upper .95
Treg	0.013	1.013	0.018	0.741	0.459	0.979	1.049
N1	2.190	8.936	1.668	1.313	0.189	0.340	234.9
N2a	2.770	15.95	1.531	1.809	0.071	0.793	320.9
N2b	4.136	62.57	1.698	2.436	0.015	2.245	1744
N3b	4.915	136.4	1.831	2.685	0.007	3.769	4933
Tfh	0.203	1.225	0.169	1.197	0.231	0.879	1.706
N1	1.042	2.835	2.011	0.518	0.604	0.055	146.0
N2a	3.264	26.15	1.578	2.068	0.039	1.187	576.3
N2b	4.649	104.4	1.836	2.532	0.011	2.858	3816
N3b	5.595	269.0	2.027	2.760	0.006	5.062	14297
Th2	0.120	1.127	0.064	1.868	0.062	0.994	1.278
N1	5.994	400.9	2.878	2.082	0.037	1.423	113000
N2a	2.510	12.30	1.638	1.532	0.126	0.496	305.1
N2b	6.029	415.1	2.550	2.365	0.018	2.805	61420
N3b	6.538	690.6	2.560	2.553	0.011	4.569	104400

Note: coef: estimated coefficients; HR: hazard ratio; se(coef): standard error of the coefficient; Treg: regulatory T cell; Tfh: T follicular helper cell; Th2: T helper 2 cell.
